# A comparison of the enzymatic properties of three recombinant isoforms of thrombolytic and antibacterial protein—Destabilase-Lysozyme from medicinal leech

**DOI:** 10.1186/s12858-015-0056-3

**Published:** 2015-11-21

**Authors:** Alexey S. Kurdyumov, Valentin A. Manuvera, Isolda P. Baskova, Vassili N. Lazarev

**Affiliations:** Federal Research and Clinical Center of Physical-Chemical Medicine, Malaya Pirogovskaya, 1a, Moscow, 119435 Russia; Biological Faculty, M. V. Lomonosov Moscow State University, Moscow, 119991 Russia

**Keywords:** Destabilase-Lysozyme, Recombinant protein, Thrombolysis, Isopeptidase, Antimicrobial activity

## Abstract

**Background:**

Destabilase-Lysozyme (mlDL) is a multifunctional i-type enzyme that has been found in the secretions from the salivary glands of medicinal leeches. mlDL has been shown to exhibit isopeptidase, muramidase and antibacterial activity. This enzyme attracts interest because it expresses thrombolytic activity through isopeptidolysis of the ε-(γ-Glu)-Lys bonds that cross-link polypeptide chains in stabilised fibrin. To date, three isoforms of mlDL have been identified.

The enzymatic properties of pure mlDL isoforms have not yet been described because only destabilase complexes containing other proteins could be isolated from the salivary gland secretion and because low product yield from the generation of recombinant proteins has made comprehensive testing difficult.

**Results:**

In the present study, we optimised the procedures related to the expression, isolation and purification of active mlDL isoforms (mlDL-Ds1, mlDL-Ds2, mlDL-Ds3) using an *Escherichia coli* expression system, and we detected and compared their muramidase, lytic, isopeptidase and antimicrobial activities. After optimisation, the product yield was 30 mg per litre of culture. The data obtained in our study led to the suggestion that the recombinant mlDL isoforms isolated from inclusion bodies form stable oligomeric complexes. Analyses of the tested activities revealed that all isoforms exhibited almost identical patterns of pH and ionic strength effects on the activities. We determined that mlDL-Ds1, 2, 3 possessed non-enzymatic antibacterial activity independent of their muramidase activity. For the first time, we demonstrated the fibrinolytic activity of the recombinant mlDL and showed that only intact proteins possessed this activity, suggesting their enzymatic nature.

**Conclusions:**

The recombinant Destabilase-Lysozyme isoforms obtained in our study may be considered potential thrombolytic agents that act through a mechanism different from that of common thrombolytics.

**Electronic supplementary material:**

The online version of this article (doi:10.1186/s12858-015-0056-3) contains supplementary material, which is available to authorized users.

## Background

The use of blood-sucking leeches for medical purposes in humans has been known since ancient times [1, 2]. The leeches were mainly used for bloodletting because evolutionary adaptations of their feeding apparatus promote the inhibition of haemostasis and blood coagulation [3–5]. The composition of leech salivary gland secretions plays a major role in this inhibition [3, 6–8]. Moreover, in 1948, the salivary gland secretions of leeches were shown to have thrombolytic activity [9]. Decades later, in 1984, Baskova and Nikonov revealed that destabilised fibrin depolymerisation occurred after the application of leech salivary gland secretions on stabilised fibrin plates only [6]. Moreover, the process of fibrinolysis held better on a more stabilized fibrin by factor XIIIa. The authors proposed that leech salivary gland secretions contained an enzyme that caused fibrin depolymerisation via the hydrolysis of ε-(γ-Glu)-Lys isopeptide bonds between polypeptide chains in stabilised fibrin. Later, the enzyme that possessed thrombolytic activity was isolated and identified. This enzyme was referred to as destabilase [10].

To demonstrate the isopeptidase activity of destabilase, a synthetic analogue of the dipeptide L-γ-glutamine-p-nitroanilide (L-γ-Glu-pNA) [11] and D-dimer (the final proteolytic degradation product of stabilised fibrin) [12] were used. ε-(γ-Glu)-Lys bonds cross-link D-monomers in the D-dimer. Destabilase has been shown to exhibit isopeptidase activity in relation to D-dimers without degrading the fibrinogen and serum albumin [13]. This mechanism was revealed through an analysis of the N-terminal amino acid sequence of the γ-chain in the D-monomer after isopeptidolysis of ε-(γ-Glu)-Lys bonds [14].

The thrombolytic activity of destabilase has also been demonstrated in an animal (rat) model with a pre-formed thrombus. The thrombus weight was been found to decrease by 85 and 98 % at 48 and 137 h, respectively, after the intravenous injection of destabilase [15].

In 2000, a comparison of the amino acid sequence of destabilase with the sequences of other known proteins revealed the similarity of its fragment to *Asteria rubens* lysozyme, suggesting that destabilase possesses muramidase activity [16]. Muramidase activity involves the hydrolysis of the β-(1,4)-glycoside bond between N-acetylmuramic acid and N-acetylglucosamine in the bacterial peptidoglycan [17]. The muramidase activity of destabilase was demonstrated by the cell wall disruption of *Мicrococcus lysodeikticus* [16]. At the same time, this enzyme with heat-inactivated muramidase activity still exhibited high antimicrobial activity against not only bacteria but also yeasts, fungi, and archaea [13, 18, 19]. It has also been shown that synthetic amphipathic fragments of the destabilase possess antimicrobial activity [18]. Thus, destabilase was the first multifunctional i-type invertebrate lysozyme to be discovered [16], later called Destabilase-Lyzosyme (mlDL) [20]. At the moment, there is only a three-dimensional model of mlDL-Ds2 based on the crystal structure of a highly homologous lysozyme from *Tapes japonica* (TJL) [21]. Among the proteins for which the spatial structure was identified, the primary structure of TJL is mostly similar to the primary structure of destabilase (identity ~46 %). Based upon indirect observations, it wassuggested that two distinct active centres in the molecule of TJL are responsible for its isopeptidase and lysozyme activities [22]. 3D model of mlDL-Ds2 structure showed that both functional centers are located relatively close to each other with their overlapping [20].

Because of its properties, mlDL could serve as a potential thrombolytic agent with a low rate of thrombolysis [13].

Previous studies have described the enzymatic properties of mlDL purified from leech salivary gland secretions [10, 16, 23, 24]. The isolation and purification of mlDL directly from the salivary gland secretions of medicinal leeches is a laborious process with a low yield of the final product. Moreover, mlDL is isolated from the salivary gland secretions in the form of a destabilase complex that includes hirudin, 6-keto prostaglandin F1α and plasma kallikrein inhibitor [23]. Alternatively, recombinant mlDL is used to study the properties of the pure protein. To date, three genes (*Ds-1, −2, −3*) have been identified that encode three isoforms of the destabilase (Ds-1, −2, −3) [25, 26]. All isoforms were found in *H. medicinalis*. Ds-1 and Ds-2 were found by mRNA analysis, Ds-3 was found by sequencing of CnBr-fragments of destabilase isolated from leech’s saliva. The homology between mlDL isoforms varied from approximately 66 to 87 % (Additional file 1: Figure S1). Previously, only two recombinant isoforms of mlDL were expressed in *Escherichia coli* (mlDL-Ds2, 3) [27]. Moreover, mlDL-Ds2 was obtained in a *Spodoptera frugiperda* baculovirus system [28]. Nevertheless, the low final product yield impeded the study of the properties of the pure enzyme. The goal of our study was to generate active mlDL isoforms and compare their enzymatic properties. To this end, we constructed plasmids with sequences that encoded all of the mlDL isoforms. We developed the methods for the expression, isolation and purification of the active mlDL isoforms and detected and compared their lysozyme (muramidase and lytic), isopeptidase and antimicrobial activities. Additionally, to test whether the mlDL isoforms possess antibacterial activity independent of their enzymatic activities, tryptic peptides were generated.

## Methods

### Generation of the recombinant mlDL isoforms in *E. coli*

DNA fragments that encode the mlDL-Ds1, 2, 3 [25] were *de novo* synthesised from oligonucleotides. These fragments were optimised for expression in *E. coli*. A list of the oligonucleotides and a scheme for synthesising the fragments are presented in Additional file 2: Table S1 and Additional file 3, respectively. The commercially available plasmid pET-15b (Novagen, USA) was modified by adding new multiple cloning sites. The resulting plasmid was termed pET15MCS (Additional file 3: Figure S2). The DNA fragments encoding the mlDL isoforms and plasmid pET15MCS were treated with the restriction endonucleases BamHI and SalI. The restriction products were ligated. Prepared plasmids were subsequently used for the standard transformation procedure of *E. coli* Top10 cells. PCR selection of the colonies was performed using the oligonucleotides T7 and T7t. Next, the plasmids were extracted and sequenced. Because of the identity of the plasmids that encoded the three mlDL isoforms, we termed them pET15/Dest. A map of the pET15/Dest plasmid is shown in Additional file 3: Figure S3. The *E. coli* strain BL21(DE3)-gold was transformed by the plasmids that encoded the three mlDL isoforms. The transformed cells were plated on a selective solid medium containing ampicillin (150 ng/ml) and then incubated at 37 °C for 16 h. Individual colonies of *E. coli* were inoculated into super broth (SB) medium containing potassium nitrate (2 g/l) and cultured at 37 °C overnight. The cultures were then added to the fresh SB medium containing potassium nitrate at a 1:20 dilution, and the cells were grown until optical density OD_600_ = 0.8. Next, lactose was added to a final concentration of 10 mM, and the cultures were incubated again at 37 °C for 6 h. Inclusion bodies were then isolated and solubilised in denaturing buffer containing 8 M urea. The mlDL-Ds1, 2, 3 were isolated using metal chelate affinity chromatography under denaturing conditions (for details, see Additional file 4).

The purified mlDL isoforms were diluted in 8 M urea at a final protein concentration below 1 mg/ml and renatured using stepwise dialysis against the solution containing 20 mM NaH_2_PO_4_, 150 mM NaCl with the following decreasing urea concentration: 4, 2, 1, and 0 M. Each step lasted at least 8 h. The precipitate was separated by centrifugation at 10,000 × g for 20 min.

### MALDI-TOF analysis

The protein bands after 1D PAGE were subjected to trypsin ingel hydrolysis. Gel pieces (2 mm^3^) were excised and washed twice with 100 ml of 0.1 M NH_4_HCO_3_ and 40 % acetonitrile for 30 min at 37 °C, dehydrated with 100 ml of acetonitrile and air-dried. Then, they were treated with 4 ml of a 12.5 mg/ml solution of modified trypsin (Promega) in 40 mM NH_4_HCO_3_ and 10%acetonitrile for 16 h at 37 °C. The peptides were extracted with 8 ml of an aqueous solution of 0.5 % trifluoroacetic acid for 20 min. Aliquots (2 ml) of the sample were mixed on a steel target with 0.3 ml of a 2,5-dihydroxybenzoic acid (Brucker Daltonics, Germany) solution (75 mg/ml in 30 % acetonitrile/0.5 % trifluoroacetic acid). Mass spectra were recorded on an Ultraflex II MALDI-ToFToF mass spectrometer (Brucker Daltonics, Germany) equipped with an Nd laser. The [MH^+^] molecular ions were measured in reflector mode; the accuracy of the mass peak measurement was 0.005 %. The fragment ion spectra were generated by laser-induced dissociation slightly accelerated by low-energy collision-induced dissociation using helium as the collision gas. The accuracy of the fragment ion mass peak measurement was 5 Da. The MS/MS fragments were identified using the Biotools software (Brucker Daltonics, Germany) and Mascot MS/MS ion search. Protein identification was performed using a peptide fingerprint search with the Mascot software (MatrixScience Inc., USA). One missed cleavage, Met oxidation and Cys-ropionamide were permitted. Protein scores greater then 49 were supposed to be significant (*p* < 0.05).

### Buffer solutions for activity assays (standard buffers)

We assessed the pH effect on the enzymatic activities using the following standard buffers [29]: citric acid/sodium citrate buffer for the pH range of 1.0–5.0, sodium citrate/sodium phosphate buffer for the pH range of 5.0–7.0, sodium phosphate/sodium hydroxide buffer for the pH range of 7.0–12.0, and potassium chloride/sodium hydroxide buffer for the pH range of 12.0–13.0. The total concentration of the components in each buffer was kept at 10 mM. After determining the optimal pH conditions that corresponded to the activity peaks, we assessed the ionic strength effect on the enzymatic activities at the predetermined optimal pH conditions. In this case, the ionic strength of the solutions varied with the concentration of NaCl.

### Muramidase activity assay

Muramidase activity was determined via clarifying the suspension of lyophilised *M. lysodeikticus* cells (0.5 mg/ml; Sigma, USA) after treatment with the mlDL isoforms [30, 31]. Hen egg white lysozyme (HEWL; Sigma, USA) was used as a reference lysozyme with known muramidase activity under the definite experimental conditions: at 25 °C in 20 mM Na-phosphate buffer, pH 7.4. The concentrations of both mlDL isoforms and HEWL varied from 0 to 20 μg/ml, and the sample volume was 200 μl. Incubation took place in the wells of a 96-well plate at 25 °C for 30 min. The optical densities were measured using a photometer (Multiskan Ascent, ThermoFisher Scientific, USA) at the wavelength of 405 nm. The muramidase activities of the mlDLisoforms were calculated using the following formula:

1 $$ \mathrm{A}\ \left(\mathrm{activity}\ \mathrm{units}\right) = \left({{\Delta \mathrm{O}\mathrm{D}}_{405}}^{\mathrm{d}}{{/\Delta \mathrm{O}\mathrm{D}}_{405}}^{\mathrm{h}}\right)*{\mathrm{A}}^{\mathrm{h}}, $$

where ΔOD_405_^d^ and ΔOD_405_^h^ are the differences in optical densities were measured before and after treatment with the mlDL isoforms at different pH values and HEWL at pH 7.4, respectively, and A^h^ is the known muramidase activity of HEWL at pH 7.4, which was provided by the producer (in units). Negative values that were calculated using formula (1) were set to zero.

### Lytic activity assay

Lytic activity was estimated by measuring the amount of total protein that was released from the *E. coli* (strain Top10) and *B. subtilis* (strain 186RT) cells following treatment with the mlDL isoforms. The overnight cultures of *E. coli* and *B. subtilis* were reseeded in fresh LB medium at a dilution of 1:20 and subsequently grown until OD_600_ = 1. Two hundred microlitres of the cell culture was precipitated by centrifugation at 10,000 × g for 2 min. Next, the pellet was resuspended in an equal volume of standard buffer followed by the addition of mlDL isoforms at concentrations of 20 μg/ml. The mixture was incubated at 37 °C for 30 min. Next, the sample was diluted with distilled water to a final volume of 1 ml and then centrifuged at 10,000 × g for 10 min. The supernatant was collected, and the amount of total protein released from the cells was measured using the Bradford method (QuickStart™ Bradford Protein Assay, Bio-Rad, USA) according to the manufacturer’s recommendations. It is well known that many gram-negative bacteria are lysed only in the presence of ethylenediaminetetraacetic acid (EDTA), which contributes to outer membrane disruption. Therefore, we have also determined lytic activity in the presence of EDTA (5 mM) in *E. coli*.

### Isopeptidase activity assay

Isopeptidase activity was determined by cleavage of the chromogenic substrate L-γ-Glu-pNA (Sigma, USA) following treatment with the mlDL isoforms [32]. The mlDL isoforms at concentrations of 300 μg/ml were added to standard buffers containing L-γ-Glu-pNA at a concentration of 1 mg/ml. The solutions (each sample was 200 μl) were incubated in a 96-well plate at 37 °C for 48 h. The standard buffers containing L-γ-Glu-pNA without mlDL isoforms treatment were used as control samples to exclude pH and ionic strength effects on substrate integrity. The optical densities were measured using a photometer (Multiskan Ascent) at a wavelength of 405 nm. The isopeptidase activity was calculated using the following formula:

2 $$ \mathrm{A}\ \left(\mathrm{activity}\ \mathrm{units}\right) = \left({{\Delta \mathrm{O}\mathrm{D}}_{405}}^{\mathrm{d}}{{\hbox{--} \Delta \mathrm{O}\mathrm{D}}_{405}}^{\mathrm{c}}\right)*1000, $$

where ΔOD_405_^d^ and ΔOD_405_^c^ are the differences in the optical densities, which were measured before and after treatment with mlDL isoforms for the experimental and control samples, respectively, at the same pH values. Negative values that were calculated using formula (2) were set to zero.

### Preparation of tryptic peptides from the mlDL isoforms

Trypsin (0.1 μg/ml; Gibco®, USA) was added to the solutions containing mlDL isoforms at the concentration of 200 μg/ml. The solutions were incubated at 37 °C for 1 h. Next, trypsin was inhibited by adding phenylmethanesulfonylfluoride (PMSF; Sigma, USA) at a concentration of 1 mM, and the solutions were subsequently incubated at room temperature for additional 3 h. We used a refolding buffer (20 mM NaH_2_PO_4_, 150 mM NaCl) that had undergone the same preparation procedures as a control solution.

### Antibacterial activity assay

The antibacterial activity of the mlDL isoforms and their tryptic peptides was independently determined using the following two methods:

Method 1. Determination of the growth curves of bacteria treated with the mlDL isoforms and their tryptic peptides. The overnight cultures of *E. coli* and *B. subtilis* cells were inoculated into LB medium at a dilution of 1:20 and then the mlDL isoforms or their tryptic peptides were added at final concentrations varying from 0 to 20 μg/ml along with the twofold dilutions. The cells were subsequently grown in a 96-well plate at 37 °C for 7 h. The sample volume was 200 μl. The optical density (OD_600_) was measured every 30 min for each sample at a wavelength of 600 nm using a photometer. The total observation time was 7 h for each sample. Next, the growth curves were expressed as the time dependence of OD_600_.

Method 2. Determination of the minimum inhibitory concentrations (MICs) of the mlDLisoforms and their tryptic peptides [33]. A total of 150 μl of LB medium containing 5*10^5^ CFU/ml *E. coli* or *B. subtilis* was inoculated into each well of a 96-well plate. The cells were incubated with mlDL isoforms at 37 °C for 16–18 h, and their tryptic peptides at the concentrations were varied from 0 to 20 μg/ml. Next, the samples were plated onto solid LB medium in Petri dishes. The Petri dishes were incubated at 37 °C for 18 h. The growth colonies were counted, and the MICs were determined.

### Fibrinolytic activity assay

Fibrinolytic activity was assayed using the fibrin plate method [34] with a slight modification. Briefly, fibrin plates were prepared by the addition of 6 mg of fibrinogen (Tehnologia-Standart, Russia) and 0.5 U of thrombin (Tehnologia-Standart, Russia) to 15 ml of 0.1 M Na-phosphate buffer (pH 7.4) containing 0.15 M NaCl. The resulting solution was immediately inoculated onto a 90-mm Petri dish. Fibrin gel formation continued at room temperature for 4 h. Droplets (5 μl) of the solutions containing mlDL isoforms at the concentration of 1 mg/ml were carefully placed on the plate and incubated at room temperature for 48 h. We measured the diameters of the lysed zones produced by each droplet. The measurements of each lysed zone were performed in three directions followed by the calculation of the mean value. The experiments were repeated independently in three fibrin plates. Percentage of stabilized fibrin in fibrin clot was determined according to the amount of soluble protein in 2 % acetic acid [35]. Unstabilized fibrin is dissolved in acid, stabilized fibrin is precipitated.

### Circular dichroism

The CD spectra were recorded using a Chiroscan CD spectrophotometer (Applied Photophysics, UK). The spectra weremeasured between 180 and 280 nm (1 nm step) at 20 °C. A 2 mm-pathlength cell with a detachable window was used. The protein concentrations were 2 mg/ml in the buffer (5 mM Na_2_HPO_4_, pH 2.2, 3.2 and 6.5).

## Results

### Generation of the active recombinant mlDL isoforms in *E. coli*

**Fig. 1:**
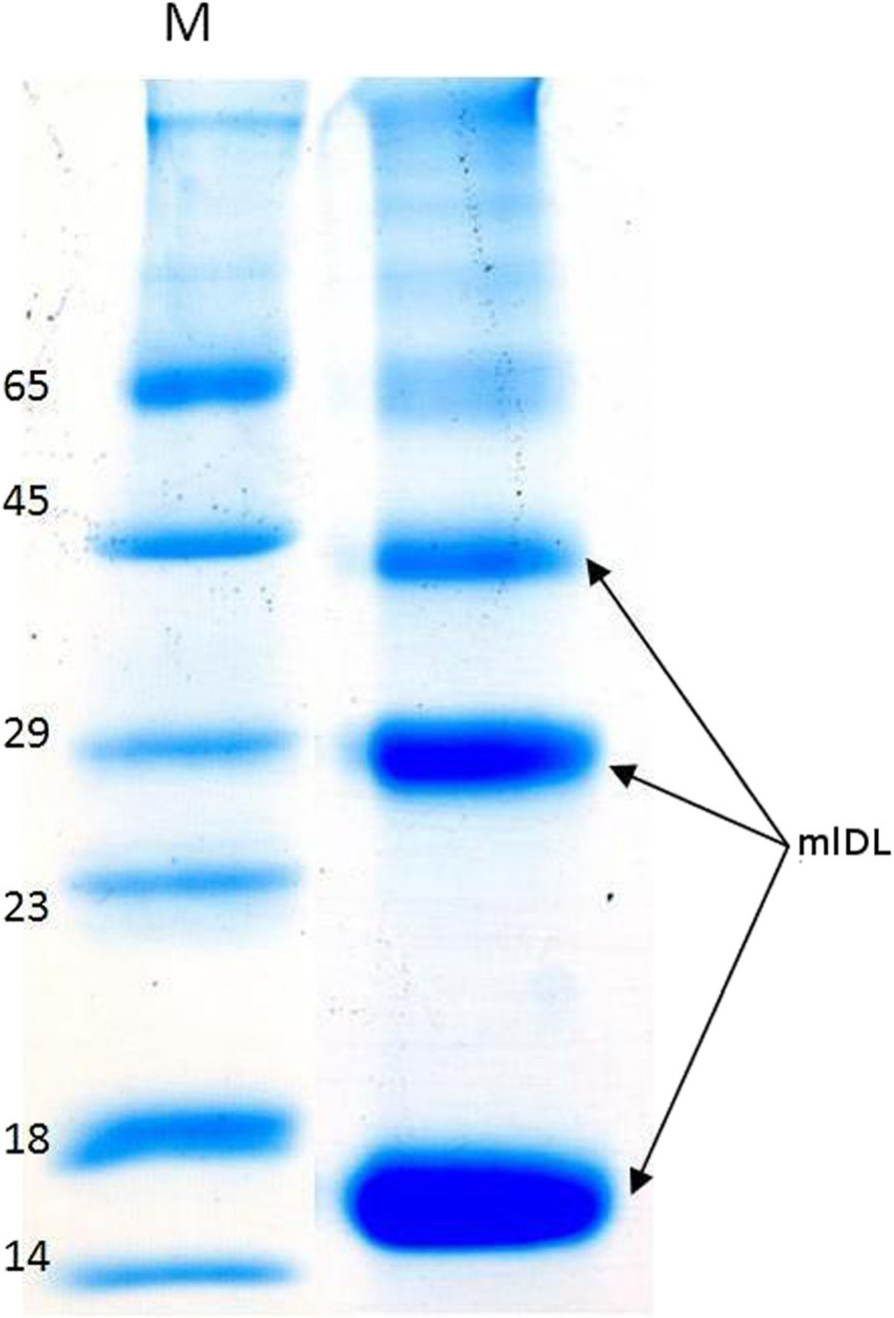
SDS-PAGE of mlDL-Ds3. mlDL-Ds3 was isolated using metal chelate affinity chromatography. mlDL-Ds3 was heating 10 min at 95 °C with 2 % 2-mercaptoethanol. M—molecular weight marker (kDa). Electrophoresis was performed in 13.5 % acrylamide/bis gel. The gel was stained with Coomassie 250G

To optimise the accumulation of the mlDL isoforms in *E. coli* cells, we empirically selected the strains (8 strains), media (7 media) and cultivation schemes. All optimisation procedures and an optimal refolding scheme and cultivation method are described in Additional file 4. The final yields of the active mlDL isoforms were 25–30 mg per 1 litre of the bacterial culture. An electrophoregram of the isolated mlDL-Ds3 is shown in Fig. 1. Well-separated bands with molecular weights of approximately 16, 28 and 44 kDa were detected. Using MALDI-TOF analysis we confirmed that these bands consisted of mlDL (Additional file 5. Figure S4). Thus, we supposed that the recombinant mlDL-Ds3 forms stable oligomeric complexes. Similar results were obtained for other mlDL isoforms, mlDL-Ds1 and mlDL-Ds2 (data not shown). But by gel-filtration chromatography on Sephadex 200 mlDL isoforms form only one peak corresponding to a monomer of mlDL (Additional file 6: Figure S5).

### Muramidase activity of the mlDL isoforms

**Fig. 2:**
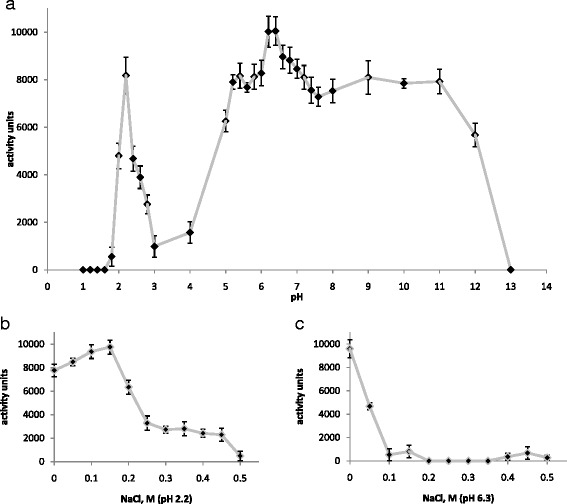
Muramidase activity of mlDL-Ds3. Effects of pH (**a**) and ionic strength (**b**, **c**) on the muramidase activity of mlDL-Ds3. The activity (in units) was calculated relative to the reference enzyme HEWL according to formula (1). (*n* = 5)

We determined the pH effect on the muramidase activity of the mlDL isoforms by clarifying the suspension of lyophilised *M. lysodeikticus*. All measurements were conducted in the linear range of the decrease of absorbance M. lysodeikticus suspension before reaching the saturation level. It is shown in Additional file 7 at pH 6.3 without NaCl. Figure 2a shows the results of the assay for mlDL-Ds3. mlDL-Ds3 exhibited muramidase activity at a wide pH range of 5.0–12.0, with a peak at pH 6.3. We also detected an extremely high activity peak at pH 2.2. Subsequently, we analysed the effect of ionic strength on muramidase activity under these optimal pH conditions. mlDL-Ds3 exhibited the greatest activity at pH 2.2 with the NaCl concentration adjusted to 150 mM (Fig. 2b) and at pH 6.3 without NaCl addition (Fig. 2c). Similar results were obtained for other mlDL isoforms, mlDL-Ds1and mlDL-Ds2 (Additional file 8: Figure S7). The muramidase actibity of mlDL isoforms at the pH 6.3 without NaCl performed in the Table 1.

**Table 1:** Comparison of the mlDL fermentative activities and half-maximal diapasones

	Muramidase activity (in lysozyme units)	Lytic activity	Isopeptidase activity			Half-maximal pH and I values for		
			in activity units	K_m_, μM	k_kat_, ×10^−2^ s^−1^	muramidase activity	lytic activity	isopeptidase activity
mlDL-Ds1	5200 ± 450	0. 079 ± 0.005	850 ± 70	40	7	pH 2.5–3, I = 0–0.25 M;	pH 4.5–10	pH 4.5–7.5
mlDL-Ds2	2450 ± 250	0. 035 ± 0.003	660 ± 60	50	8	pH 4.5–12, I = 0–0.05 M	I = 0–0.4 M	I = 0–0.1 M
mlDL-Ds3	10,000 ± 600	0. 136 ± 0.005	1780 ± 160	20	5			

### Lytic activity of the mlDL isoforms

**Fig. 3:**
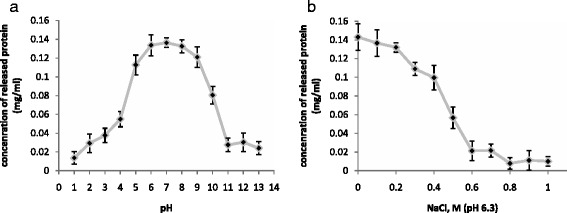
Lytic activity of mlDL-Ds3. Effects of pH (**a**) and ionic strength (**b**) on the lytic activity of mlDL-Ds3. The activity was expressed as the concentration of protein released from the cells of *B. subtilis*. (*n* = 5)

We determined the pH effect on the lytic activity of the mlDL isoforms by measuring the amount of total protein that was released from the gram-positive *B. subtilis* and gram-negative *E. coli* cells. mlDL-Ds3 exhibited lytic activity against *B. subtilis* at the pH range of 5.0 to 9.0 (Fig. 3a). In this pH range, mlDL-Ds3 possessed almost equal activity values. Therefore, to determine the effect of ionic strength on lytic activity, we selected pH 6.3, which corresponded to the conditions in the muramidase activity assay. mlDL-Ds3 exhibited the greatest activity at NaCl concentrations ranging from 0 to 200 mM (Fig. 3b). Similar results were obtained for other mlDL isoforms, mlDL-Ds1 and mlDL-Ds2 (Additional file 9: Figure S8).

We determined the lytic activity of the mlDL isoforms against *E. coli* cells. In this case, we also tested the effect of EDTA on the lytic activity of the mlDL isoforms. It is well known that EDTA contributes to outer membrane disruption, providing access to peptidoglycan in gram-negative bacteria [36]. Peptidoglycan is known to serve as a substrate for lysozymes. The mlDL isoforms exhibited the greatest lytic activity without EDTA at the pH range of 5.0–9.0 (data not shown). In the presence of EDTA (5 mM), the greatest lytic activity slightly shifted toward alkaline conditions with the pH range of 6.0–10.0. Therefore, as for *B. subtilis*, we selected pH 6.3 to determine the effect of ionic strength on lytic activity. The mlDL isoforms exhibited the greatest lytic activity at 0 M NaCl without EDTA and at 0.3 M NaCl with EDTA (Additional file 9: Figure S8). The lytic actibity of mlDL isoforms at the pH 6.3 without NaCl and EDTA performed in the Table 1.

### Isopeptidase activities of the mlDL isoforms

**Fig. 4:**
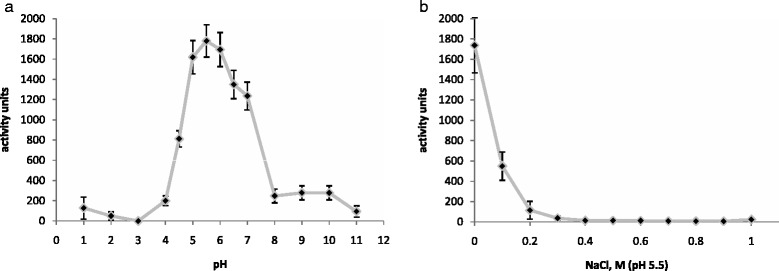
Isopeptidase activity of mlDL-Ds3. The effect of pH (**a**) and ionic strength (**b**) on the isopeptidase activity of mlDL-Ds3. The effect of ionic strength on the isopeptidase activity was determined at pH 5.5. Isopeptidase activity was calculated according to formula (2). (*n* = 5)

We determined the pH effect on the isopeptidase activity of the mlDL isoforms based on the production of p-nitroanilide L-γ-Glu-pNA, which is measured by the absorbance at 405 nm. Figure 4a shows the results of the assay for mlDL-Ds3. mlDL-Ds3 exhibited isopeptidase activity in the pH range of 4.5–7.0, with peak activity at pH 5.5. Subsequently, we analysed the effect ionic strength on isopeptidase activity at pH 5.5. mlDL-Ds3 exhibited the greatest activity without the addition of NaCl (Fig. 4b). Similar results were obtained for the other destabilase isoforms, mlDL-Ds1and mlDL-Ds2 (Additional file 10: Figure S9). The isopeptidase actibity of mlDL isoforms at the pH 5.5 without NaCl performed in the Table 1.

### Antimicrobial activity of the mlDL isoforms

We determined the antimicrobial activity of the mlDL isoforms and their tryptic peptides (mlDL-Ds1t, mlDL-Ds2t, mlDL-Ds3t) using two methods. The tryptic peptides were generated to test whether the mlDL isoforms exhibit antibacterial activity independent of their enzymatic activities. To eliminate the influence of an intact protein, we measured the muramidase activity of tryptic peptides. This mixture did not provide the enzymatic activity.

**Fig. 5:**
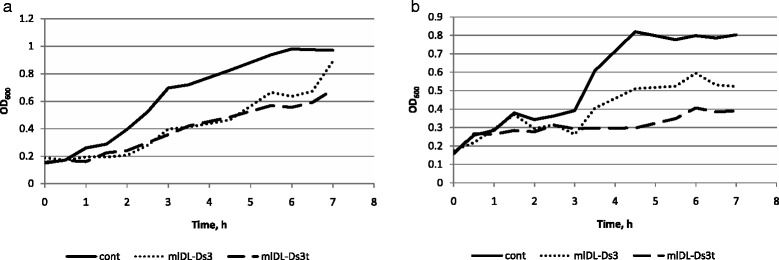
Inhibition of bacterial growth. The growth curves of the *E. coli* strain Top10 (**a**) and *B. subtilis* (**b**). cont—control cells incubated without mlDL-Ds3; mlDL-Ds3 and mlDL-Ds3t—the cells incubated in the presence of mlDL-Ds3 and mlDL-Ds3t, respectively, at a concentration of 5 μg/ml. Each growth curve was obtained by averaging three independent measurements

According to Method 1, we determined the growth curves of bacteria that were exposed to the enzymes and their tryptic peptides at different concentrations. The growth of both gram-positive and gram-negative bacteria was inhibited by mlDL isoforms and their tryptic peptides. Notably, the tryptic peptides of all mlDL isoforms exhibited more efficient bacterial growth inhibition than the whole enzymes did. Figure 5 shows the growth curves for mlDL-Ds3 and mlDL-Ds3t. Similar results were obtained for the other mlDL isoforms: mlDL-Ds1 and mlDL-Ds2 (data not shown).

In Method 2, we determined the MICs of the mlDL isoforms and their tryptic peptides (Table 2). Fewer MICs were determined for the whole mlDL isoforms and their tryptic peptides in *B. subtilis* than for *E. coli*. Moreover, for both bacterial species, the tryptic peptides had fewer MICs than the corresponding whole enzymes. These data confirm the results obtained with Method 1.

**Table 2:** Minimum inhibitory concentrations (MICs) of the recombinant mlDL isoforms and their tryptic peptides

	mlDL-Ds1	mlDL-Ds2	mlDL-Ds3	mlDL-Ds1t	mlDL-Ds2t	mlDL-Ds3t
MIC (μg/ml), *E.coli*	2.5			1.3		
MIC (μg/ml), *B.subtilis*	1.3			0.6		

### Fibrinolytic activity of the mlDL isoforms

**Fig. 6:**
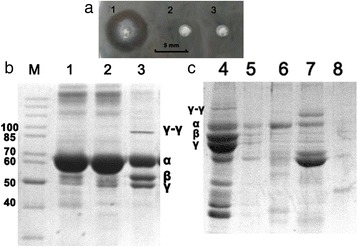
Fibrinolytic activity of mlDL-Ds3. **a** Lysed zone formation in the fibrin plate after treatment with the intact entire mlDL-Ds3. 1—mlDL-Ds3, 2—mlDL-Ds3t, 3—heat-inactivated mlDL-Ds3. **b**SDS-PAGE of fibrin gel lysed by mlDL: 1–probe from mlDL lysed zone, 2–soluble fraction of stabilized fibrin by 2 % acetic acid treatment, 3–unsoluble fraction of stabilized fibrin by 2 % acetic acid treatment, (**c**) SDS-PAGE of stabilized fibrin after mlDL-Ds3 treatment at 37 °C for 96 h: 4—unsoluble fraction of stabilized fibrin after incubation with control buffer, 5—unsoluble fraction of stabilized fibrin after incubation with mlDL-Ds3, 6—soluble fraction of stabilized fibrin after incubation with control buffer, 7—soluble fraction of stabilized fibrin after incubation with mlDL-Ds3, 8—mlDL-Ds3. M—molecular weight marker (kDa). Electrophoresis was performed in 12 % acrylamide/bis gel. The gel was stained with Coomassie 250G. α, β, γ—α, β, γ-chains of human fibrinogen (63.5, 56, 47 kDa respectively)

Fibrinolytic activity was determined by measuring the diameters of the lysed zones that resulted from the placement of droplets of the enzyme-containing solutions on fibrin plates. In this case, we investigated not only intact mlDL isoforms but also heat-inactivated (at 95 °C for 2 h) enzymes and their tryptic peptides to test whether the fibrinolytic activity was enzymatic. We determined that only the intact mlDL isoforms exhibited fibrinolytic activity. All of the intact mlDL isoforms caused lysed zones with equal diameters (6 ± 1 mm). A representative image of one such plate is shown in Fig. 6a.

Also, we performed the SDS-PAGE analysis of lysed zones (line 1, Fig. 6b). This fibrin gel was stabilized on 70 %. Solid zone of gel (untreated zone) was dissolved in 2 % acetic acid. Soluble fraction (line 2) and precipitate (dissolved in 5 % SDS, line 3) were analyzed by SDS-PAGE, too. We found the γ-γ fibrin chain only in unsoluble fraction of fibrin. This chain is absent in soluble control fraction and fraction from lysed zone by mlDL-Ds3. Then we analyzed the stabilized fibrin treated by mlDL-Ds3 (Fig. 6c). We incubated stabilized fibrin clot with mlDL-Ds3 at 37 °C for 96 h. As the control we used the buffer without mlDL. We found the γ-γ fibrin chain only in unsoluble fraction of control sample of stabilized fibrin (lane 4).

## Discussion

In this study, we obtained all known isoforms of mlDL (mlDL-Ds1, mlDL-Ds2, mlDL-Ds3) from medicinal leeches in the form of recombinant proteins in *E. coli*. We optimised the expression, isolation and purification procedures and obtained a fivefold increase in the yield of the recombinant mlDL isoforms compared with previous studies. This allowed us to investigate the enzymatic and antibacterial activities of these isoforms.

MALDI-TOF analysis data suggested that the recombinant mlDL isoforms form stable oligomeric complexes (Additional file 5: Figure S4). We have identified pure mlDL and the dimer and trimer complexes. According to gel-filtration chromatography (Additional file 6: Figure S5), mlDL forms have only one peak, corresponding to a mlDL’s-monomer. This fact does not explain the origin of these oligomeric structures. We suppose that the total protein from purified mlDL consists mostly of monomeric form, and other oligomers represent only a small part of the total protein. However observations about oligomeric forms are in agreement with previous investigations that showed that some i-lysozymes could form active dimer and trimer complexes. However, the oligomeric complexes of the destabilase isoforms were not disrupted in the solution with high ionic strength, in contrast to other i-type lysozymes [17].

Furthermore, we investigated lysozyme (muramidase and lytic), isopeptidase and antimicrobial activities of the recombinant mlDL isoforms. The greatest muramidase activity of the mlDL isoforms was detected within the pH range of 5.0–12.0. Surprisingly, we also detected the peak of the muramidase activity at extremely acidic pH 2.2. Most of the known i-type lysozymes exhibit the greatest muramidase activity within the pH range of 6.0–8.0 [17], but these lysozymes have not yet been shown to possess activity at the extremely acidic pH. We have analysed the circular dichroism (CD) spectra of mlDL isoforms at different pH conditions: 2.2 and 6.5 are corresronds the maximum of muramidase activity, 3.2—minimum of muramidase activity (Additional file 11: Figure S10). All probes of the mlDL exhibited similar CD spectra. However, the spectra are shifted to the long wavelength waves with increasing pH to all isoforms of mlDL. Consequently, we suppose that the conformation of the protein does not significantly affect the change in activity.

The ionic strength effect on muramidase activity differed for the different optimal pH values. The increase in NaCl concentration at pH 6.3 strongly inhibited the muramidase activity of the mlDL isoforms, a result that is in a good agreement with those obtained for mlDL isolated from the salivary gland secretion.

To test the lytic activity, we used gram-positive bacteria *B. subtilis* and gram-negative bacteria *E. coli*. The bacterial cell wall contains peptidoglycan, a main substrate for lysozyme. Peptidoglycan of gram-positive bacteria is located outside of the cell, whereas peptidoglycan of gram-negative bacteria is hidden by the outer membrane. EDTA disrupts the outer membrane in gram-negative bacteria, facilitating the enzyme’s access to peptidoglycan [36]. The mlDL isoforms exhibited the greatest lytic activity within the pH range of 5.0–9.0 for both *B. subtilis* and *E. coli*. These data are in agreement those of the previous study. In the presence of EDTA, the optimal pH range shifted toward an alkaline pH. In contrast to the muramidase activity, we have not detected the lytic activity at the acidic pH values.

The isopeptidase activity of the mlDL isoforms was determined by the production of p-nitroanilide from L-γ-Glu-pNA, which was detected by the absorbance at 405 nm. This chromogenic substrate is mainly used to study gamma-glutamyl transpeptidase and possesses a low specificity for isopeptidase. Therefore, the optimal conditions for cleavage of this substrate have been shown to require high enzyme concentrations (300–500 μg/ml) and a long incubation time (above 48 h). The mlDL isoforms exhibited isopeptidase activity in a rather narrow range of pH values (from 5.0 to 6.5). This is in agreement with the previous observations for other i-type lysozymes that possessed isopeptidase activity. We revealed that isopeptidase activity was very sensitive to the ionic strength. It has not been detected at NaCl concentrations above 0.3 M. For isopeptidase activity we measured K_m_ and k_kat_. It was found that for all isoforms K_m_ = 20–50 μM and k_kat_ = 5–8 × 10^−2^ s^−1^. So high K_m_ and low k_kat_ can be explained also by low specificity of substrate. Although these values are comparable to those obtained previously for mlDL-Ds2 (K_m_ = 50 μM, k_kat_ =0.1 × 10^−2^ s^−1^) [13] and TJL (K_m_ = 20 μM, k_kat_ = 0.2 × 10^−4^ s^−1^) [32].

For all fermentative activities we found the half-maximal pH and I values (Table 1). These values were similar for all mlDL isoforms: for lytic activity—pH 4.5–10 and I = 0–0.4 M, for isopeptidase activity—pH 4.5–7.5 and I = 0–0.1 M. For muramidase activity mlDL had two ranges: pH 2.5–3 and I = 0–0.25 M, pH 4.5–12 and I = 0–0.05 M. This information allows us to suggest the similarity of their enzymatic properties. Previous studies have demonstrated the antimicrobial activity of heat-inactivated mlDL [19]. This antimicrobial effect was also observed with synthetic amphipathic fragments of mlDL [18]. We used the mlDL isoforms and their tryptic peptides to investigate antimicrobial activity. We revealed the inhibition of bacterial growth (*E. coli* and *B. subtilis*) in the presence of the mlDL isoforms and their tryptic peptides. The tryptic peptides exhibited more efficient inhibition of bacterial growth than the entire enzymes. This was confirmed by MIC determination. The MICs of tryptic peptides were lower than those of the entire mlDL isoforms. The antimicrobial activity detected in the tryptic peptides of the mlDL isoforms confirmed that this activity is non-enzymatic. The antimicrobial activity that was independent of muramidase activity has been shown for other invertebrate lysozymes, but the mechanism of this action remains unclear.

For the first time, we have revealed that recombinant mlDL isoforms exhibit fibrinolytic activity. We determined that only the intact mlDL isoforms possess fibrinolytic activity. Neither tryptic peptides nor heat-inactivated protein exhibited fibrinolytic activity, suggesting that this activity is enzymatic and requires structural organisation of the active centre.

## Conclusions

We isolated all of the currently known mlDL isoforms from medicinal leeches and demonstrated the enzymatic activities of all of the recombinant isoforms. All of the mlDL isoforms exhibited almost identical patterns of pH and ionic strength effects on the activities. We also found that the antimicrobial activity of the mlDL isoforms is muramidase activity-independent and non-enzymatic. We found that the tryptic peptides of the mlDL isoforms exhibited more efficient inhibition of bacterial growth and had lower MICs than the whole enzymes did. We also showed that only the whole mlDL isoforms possess fibrinolytic activity.

### Availability of data and materials

The data sets supporting the results of this article are included within the article and its additional files.

## Additional files

Additional file 1: Figure S1. Comparison of amino acid sequences of mlDL isoforms. Catalytic residuesresponsible for muramidase activity are indicated with red point, and those for the isopeptidase activity with blue point. (JPG 51 kb) Additional file 2: Table S1. List of oligonucleotides used. (XLS 30 kb) Additional file 3:
**Scheme for de novo synthesising from oligonucleotides DNA fragments that encode the mlDL-Ds1, 2, 3 (optymized for** ***E. coli*** **) and map of plasmid, encoding mlDL.** **Figure S2.** Fragment of pET15MCS plasmid containing multicloning sites. PT7—promoter of late genes of bacteriophage T7; RBS—ribosome binding site; ATG—start codon; MCS—multiple cloning site; stop—stop codon; 6His—fragment encoding six histidines; tromb—fragment encoding the recognition site of thrombin. **Figure S3.** A map of the plasmid pET15/Dest. PT7—promoter of bacteriophage T7 late genes; *dest*—fragment encoding the mlDL isoforms; 6His—region encoding the hexa-histidine motive; stop—translation terminator; term—a transcription terminator; *bla*—beta-lactamase gene; pBR322ori—an origin of replication of plasmid pBR322; *lacI*—gene lacI of *E. coli*; tromb—sequence encoding the recognition site of thrombin. (PDF 119 kb) Additional file 4:
**Detailed scheme of isolating mlDL isoforms from inclusion bodies and their optimisation of renaturation by dialysis.** mlDL-Ds1, 2, 3 were isolated using metal chelate affinity chromatography under denaturing conditions. Optimisation of mlDL isoforms accumulation in *E. coli* cells. We empirically selected the strains (8 strains), media (7 media) and cultivation schemes. Table S2. Accumulation levels of the recombinant mlDL isoforms in inclusion bodies from different *E. coli* strains. Accumulation levels are expressed in mg of protein per litre of culture (*n* = 3). Table S3. Accumulation levels of the mlDL in inclusion bodies from *E. coli* BL21(DE3)-gold in different culture media. Accumulation levels are expressed in mg of protein per litre of culture (i = 3). (PDF 64 kb) Additional file 5: Figure S4. MALDI-TOF analysis of mlDL isoforms. (PDF 388 kb) Additional file 6: Figure S5. Profiles of gel-chromatography analysis of mlDL isoforms. (PDF 129 kb) Additional file 7: Figure S6. Absorbance at 405 nm of *M. lysodeikticus* suspension during mlDL-Ds3 treatment at 25 °C for 20 min in 5 mM Na-phosphate buffer pH 6.3. (*n* = 3). (PDF 50 kb) Additional file 8: Figure S7. Muramidase activity of mlDL isoforms. Effects of pH (a) and ionic strength (b, c) on the muramidase activity of mlDL-Ds1. Muramidase activity of mlDL-Ds2. Effects of pH (d) and ionic strength (e, f) on the muramidase activity of mlDL-Ds2. The activity (in units) was calculated relative to the reference enzyme HEWL according to formula (1). (*n* = 5). (PDF 106 kb) Additional file 9: Figure S8. Lytic activity of mlDL isoforms. Effects of pH (a) and ionic strength (b) on the lytic activity of mlDL-Ds1. Effects of pH (c) and ionic strength (d) on the lytic activity of mlDL-Ds2. The activity was expressed as the concentration of protein released from the cells of *B.subtilis*. Effects of pH (e) and ionic strength (f) on the lytic activity of mlDL-Ds1 in the presence of 5 mM EDTA. The activity was expressed as the concentration of protein released from the cells of *E.coli*. Effects of pH (g) and ionic strength (h) on the lytic activity of mlDL-Ds2 in the presence of 5 mM EDTA. The activity was expressed as the concentration of protein released from the cells of *E.coli*. Effects of pH (i) and ionic strength (j) on the lytic activity of mlDL-Ds3 in the presence of 5 mM EDTA. The activity was expressed as the concentration of protein released from the cells of *E. coli*. (*n* = 5). (PDF 126 kb) Additional file 10: Figure S9. Isopeptidase activity of mlDL isoforms. The effect of pH (a) and ionic strength (b) at pH 5.5 on the isopeptidase activity of mlDL-Ds1. The effect of pH (c) and ionic strength (d) at pH 5.5 on the isopeptidase activity of mlDL-Ds2. Isopeptidase activity was calculated according to formula (2). (*n* = 5). (PDF 100 kb) Additional file 11: Figure S10. CD spectra of mlDL isoforms at different pH conditions (2.2, 3.2, 6.5). (PDF 84 kb)

## Abbreviations

HEWL: Hen egg white lysozyme
MIC: Minimum inhibitory concentration
CFU: Colony-forming unit
OD: Optical density
EDTA: Ethylenediaminetetraacetic acid
PMSF: Phenylmethanesulfonylfluoride
CD: Circular dichroism
