# Supplementary figures and images for: A comparison of the enzymatic properties of three recombinant isoforms of thrombolytic and antibacterial protein—Destabilase-Lysozyme from medicinal leech
Source: BMC Biochem. 2015 Nov 21;16:27. doi: 10.1186/s12858-015-0056-3 (PMC4654880; doi:10.1186/s12858-015-0056-3)

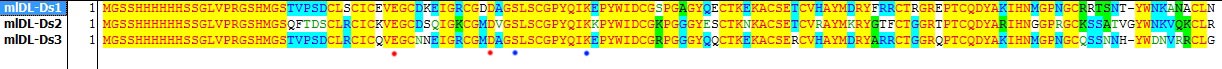

Supplement: Additional file 1: Figure S1. — Comparison of amino acid sequences of mlDL isoforms. Catalytic residuesresponsible for muramidase activity are indicated with red point, and those for the isopeptidase activity with blue point. (JPG 51 kb) [file 12858_2015_56_MOESM1_ESM.jpg]

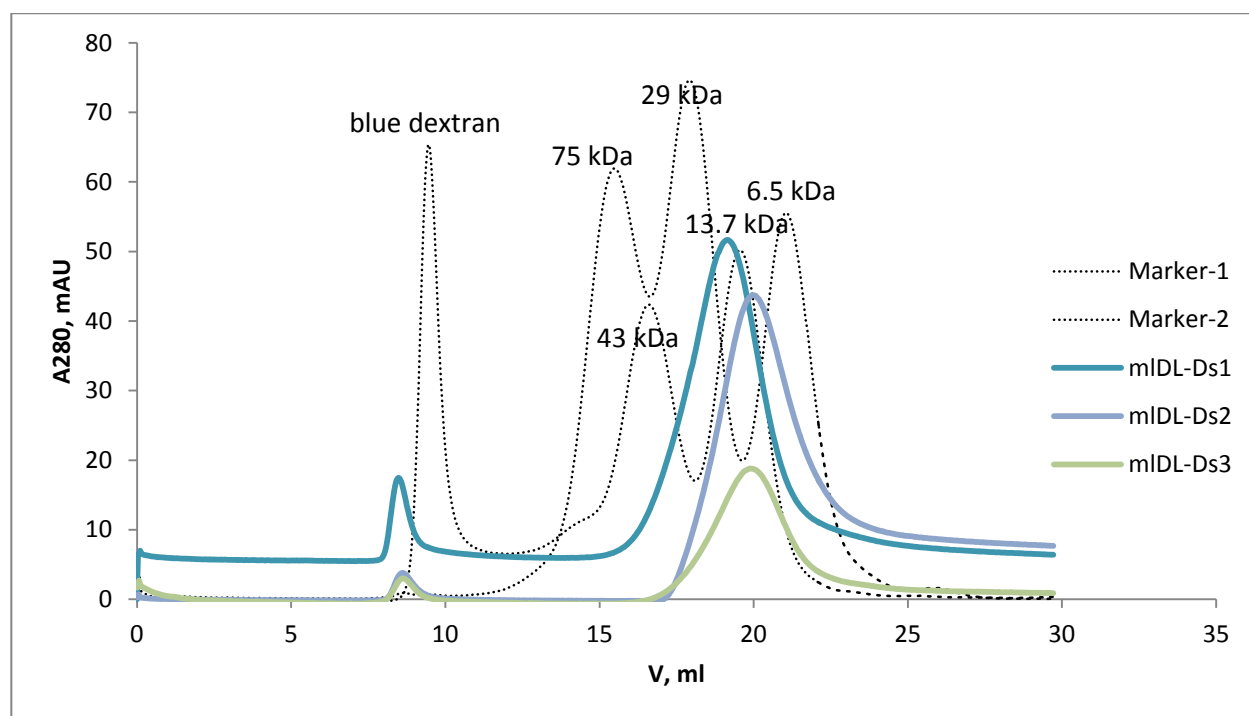

Supplement: Additional file 6: Figure S5. — Profiles of gel-chromatography analysis of mlDL isoforms. (PDF 129 kb) [file 12858_2015_56_MOESM6_ESM.pdf]

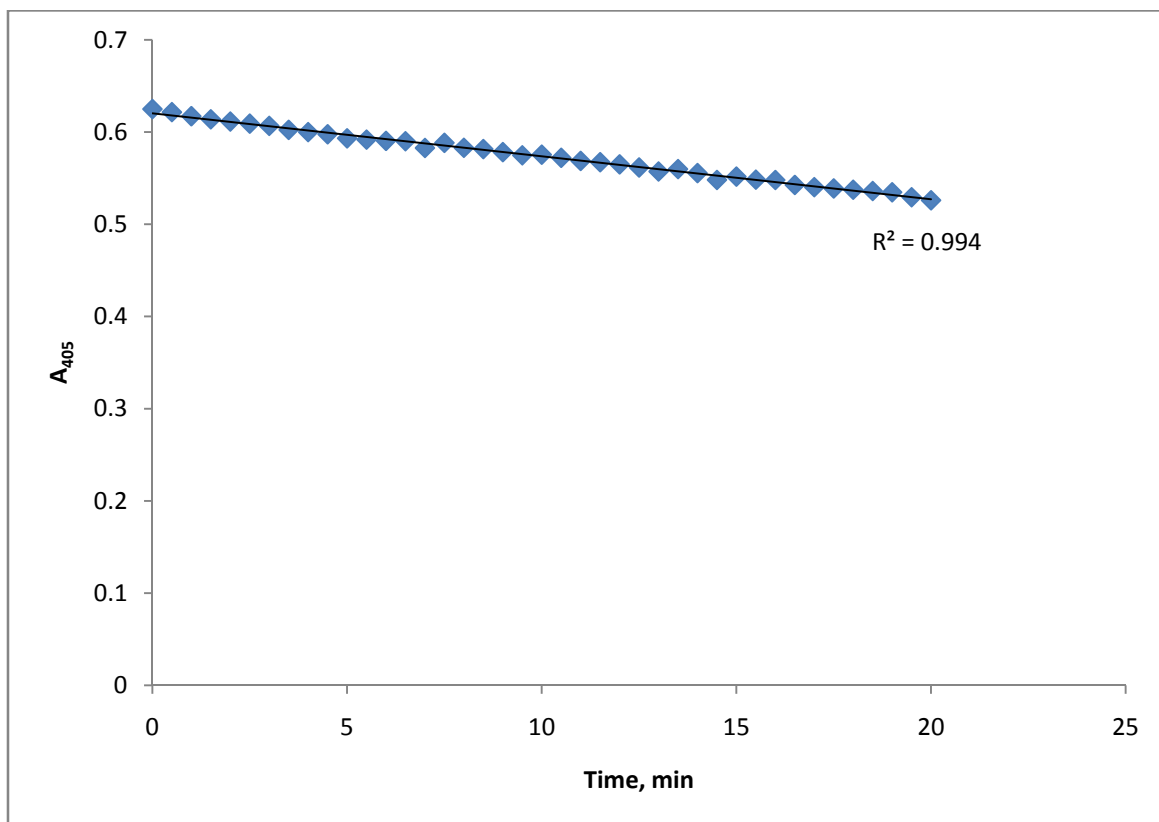

Supplement: Additional file 7: Figure S6. — Absorbance at 405 nm of M. lysodeikticus suspension during mlDL-Ds3 treatment at 25 °C for 20 min in 5 mM Na-phosphate buffer pH 6.3. (n = 3). (PDF 50 kb) [file 12858_2015_56_MOESM7_ESM.pdf]

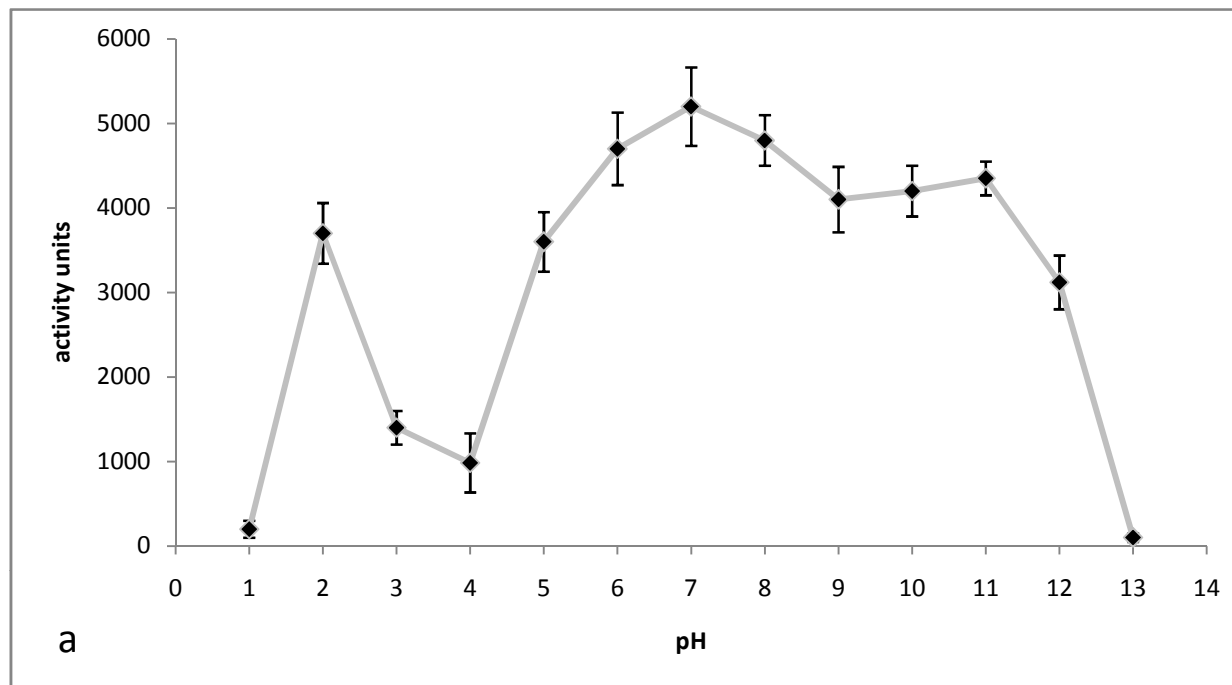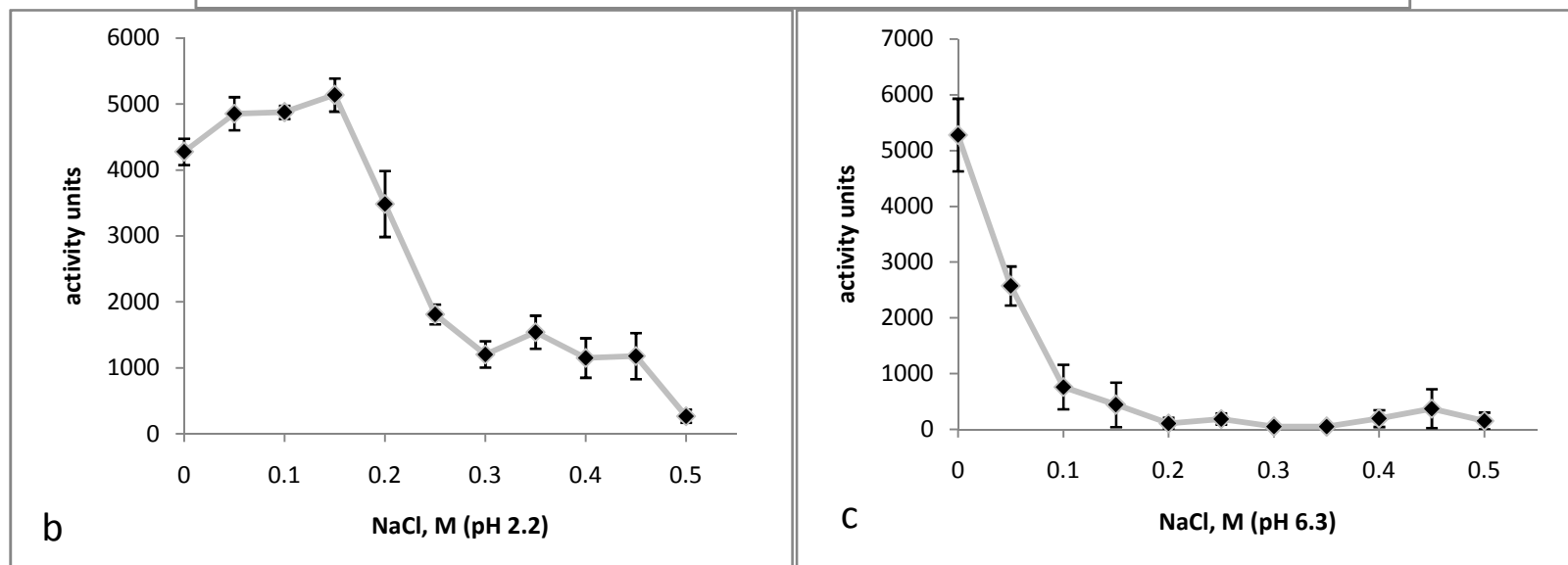

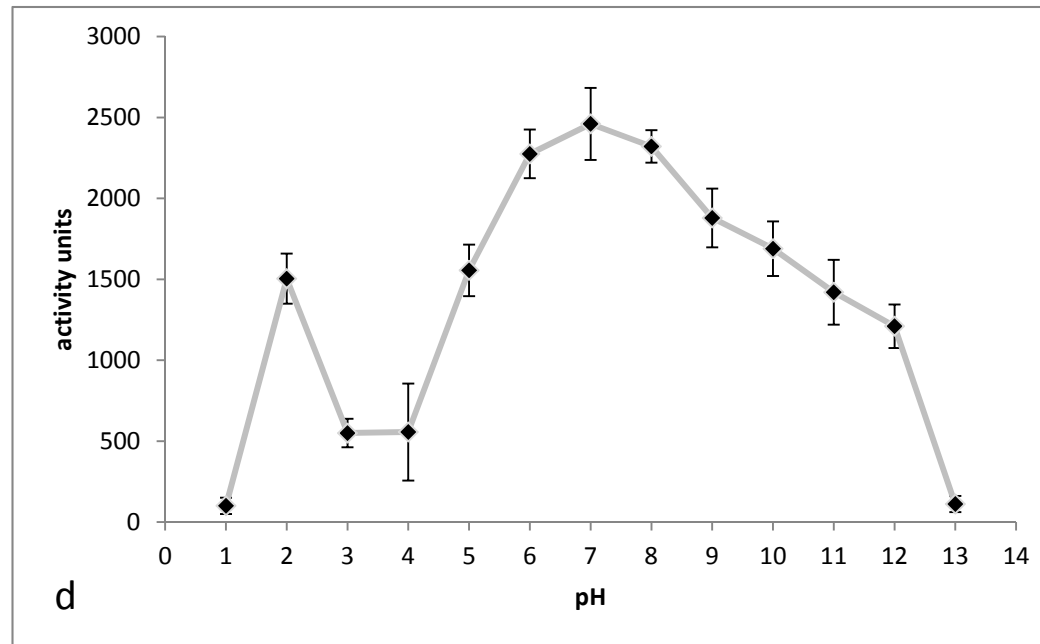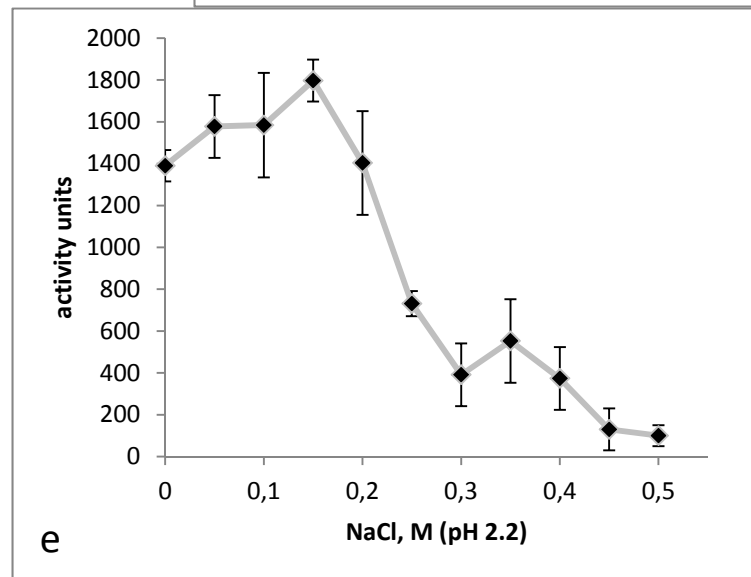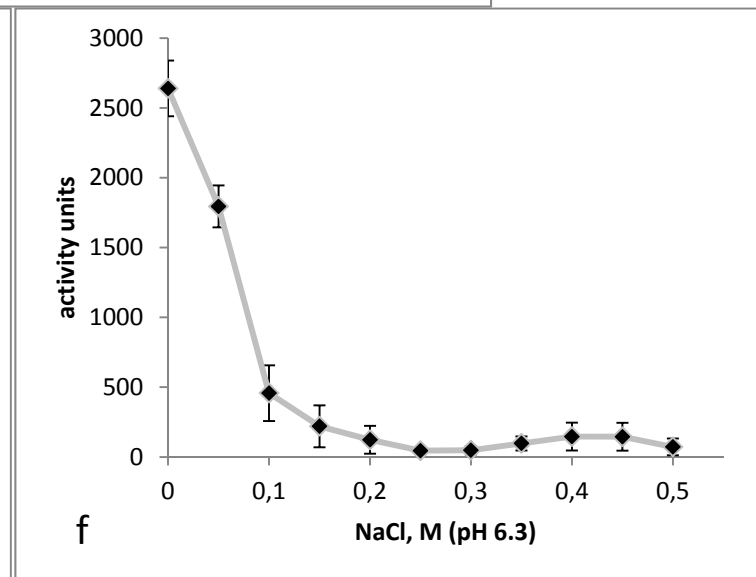

Supplement: Additional file 8: Figure S7. — Muramidase activity of mlDL isoforms. Effects of pH (a) and ionic strength (b, c) on the muramidase activity of mlDL-Ds1. Muramidase activity of mlDL-Ds2. Effects of pH (d) and ionic strength (e, f) on the muramidase activity of mlDL-Ds2. The activity (in units) was calculated relative to the reference enzyme HEWL according to formula (1). (n = 5). (PDF 106 kb) [file 12858_2015_56_MOESM8_ESM.pdf]

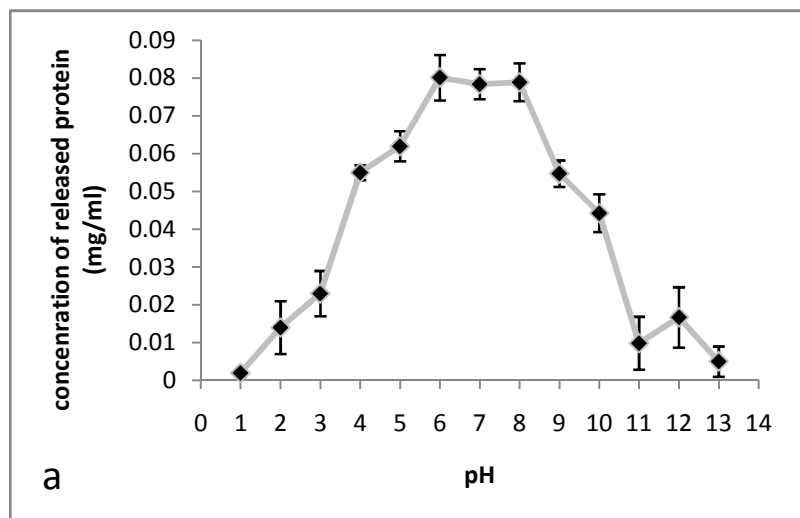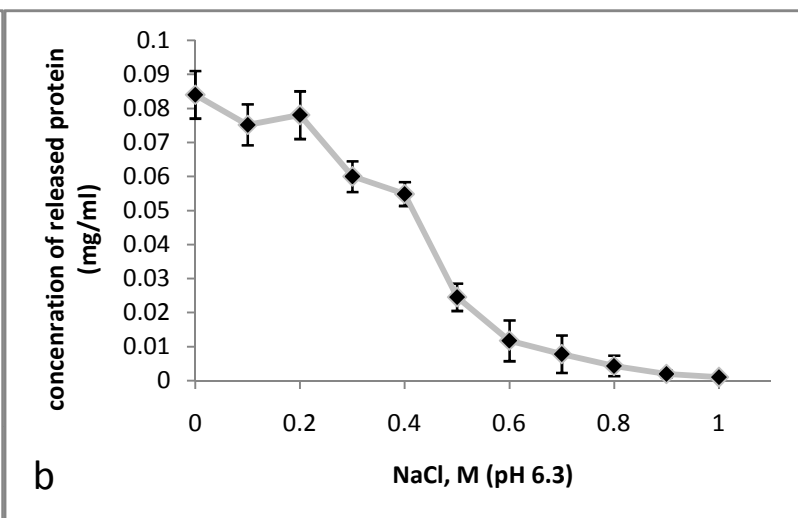

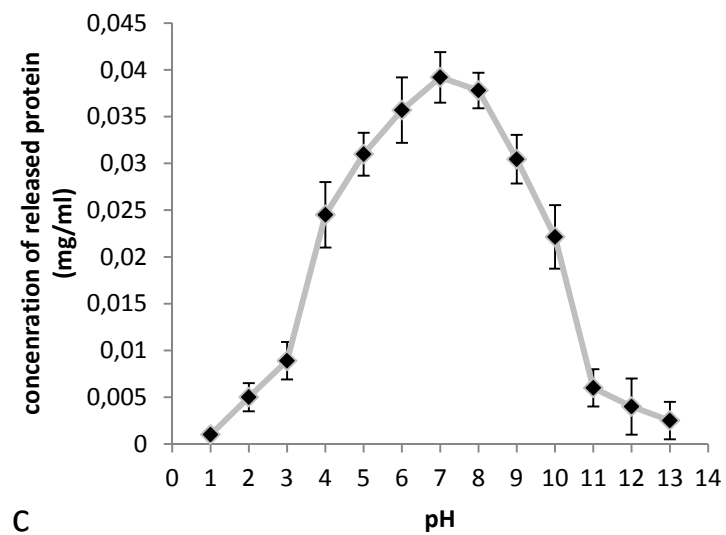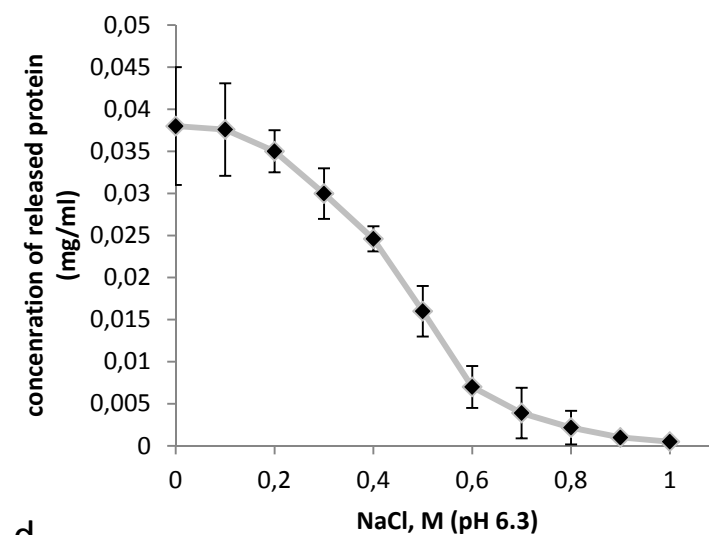

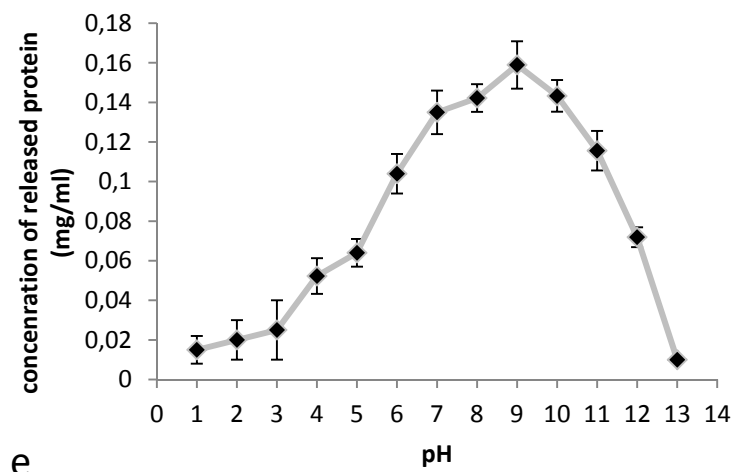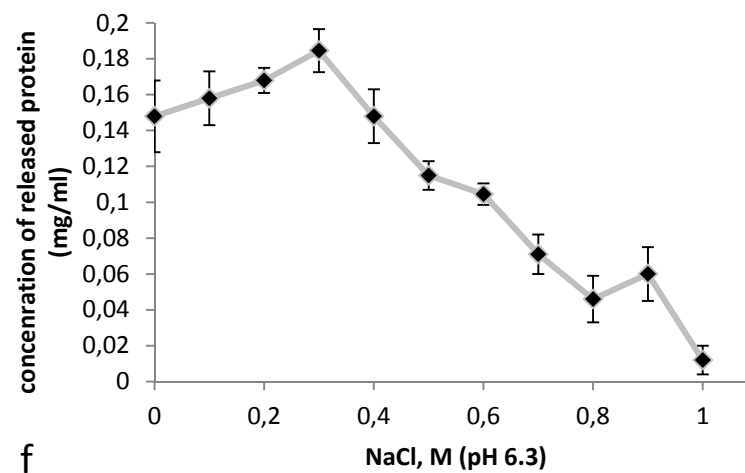

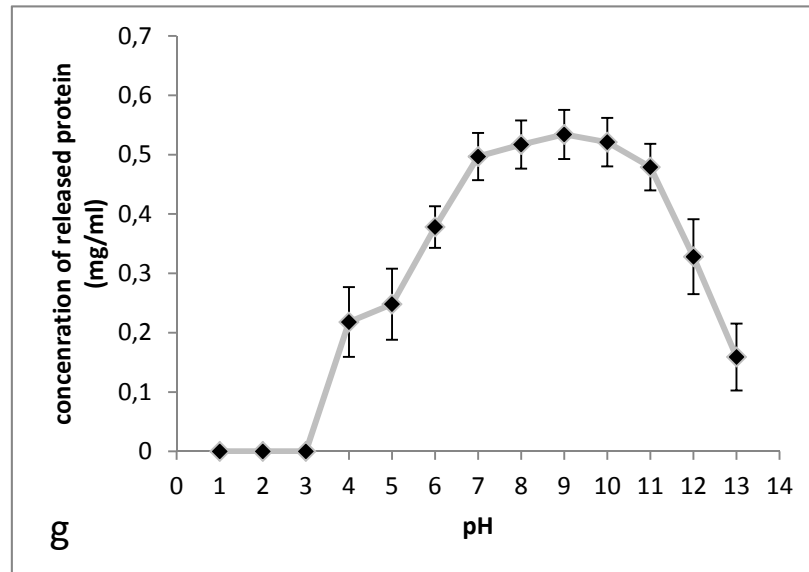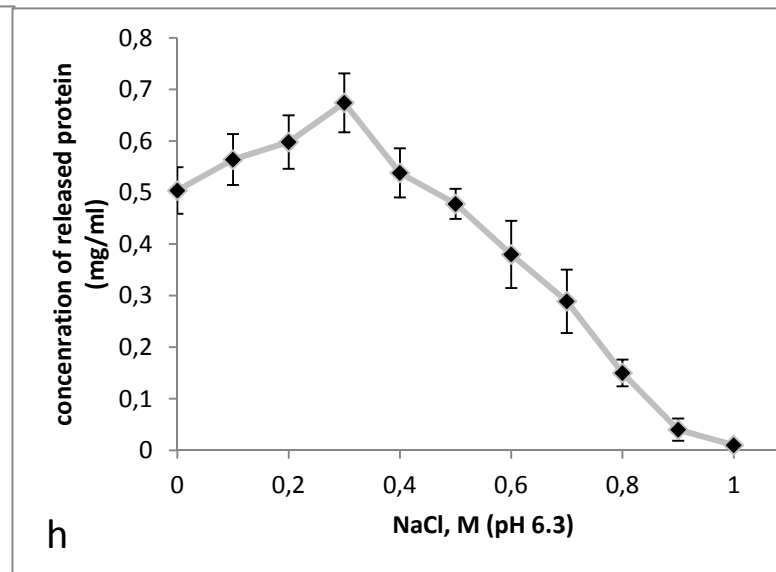

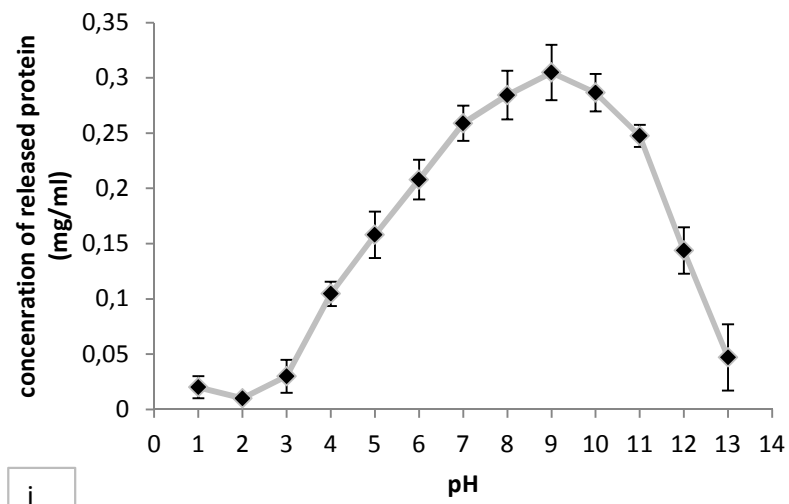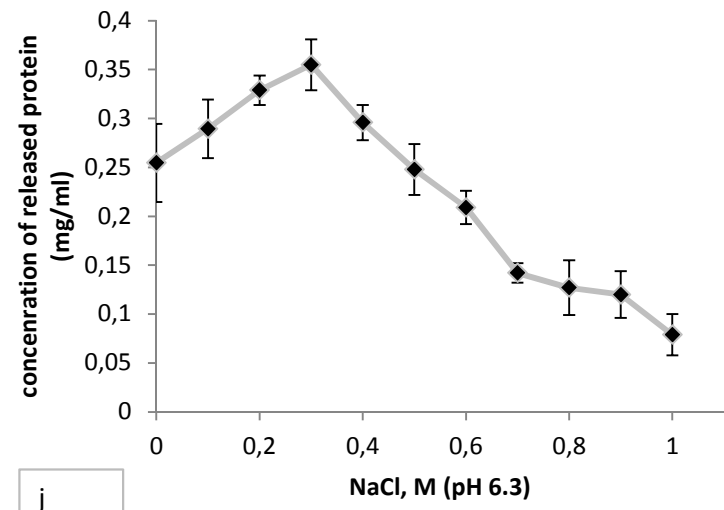

Supplement: Additional file 9: Figure S8. — Lytic activity of mlDL isoforms. Effects of pH (a) and ionic strength (b) on the lytic activity of mlDL-Ds1. Effects of pH (c) and ionic strength (d) on the lytic activity of mlDL-Ds2. The activity was expressed as the concentration of protein released from the cells of B.subtilis. Effects of pH (e) and ionic strength (f) on the lytic activity of mlDL-Ds1 in the presence of 5 mM EDTA. The activity was expressed as the concentration of protein released from the cells of E.coli. Effects of pH (g) and ionic strength (h) on the lytic activity of mlDL-Ds2 in the presence of 5 mM EDTA. The activity was expressed as the concentration of protein released from the cells of E.coli. Effects of pH (i) and ionic strength (j) on the lytic activity of mlDL-Ds3 in the presence of 5 mM EDTA. The activity was expressed as the concentration of protein released from the cells of E. coli. (n = 5). (PDF 126 kb) [file 12858_2015_56_MOESM9_ESM.pdf]

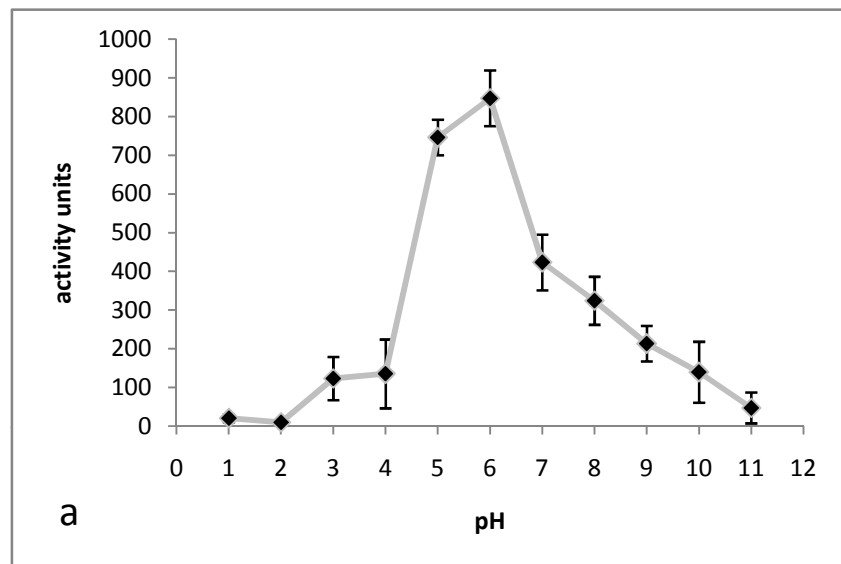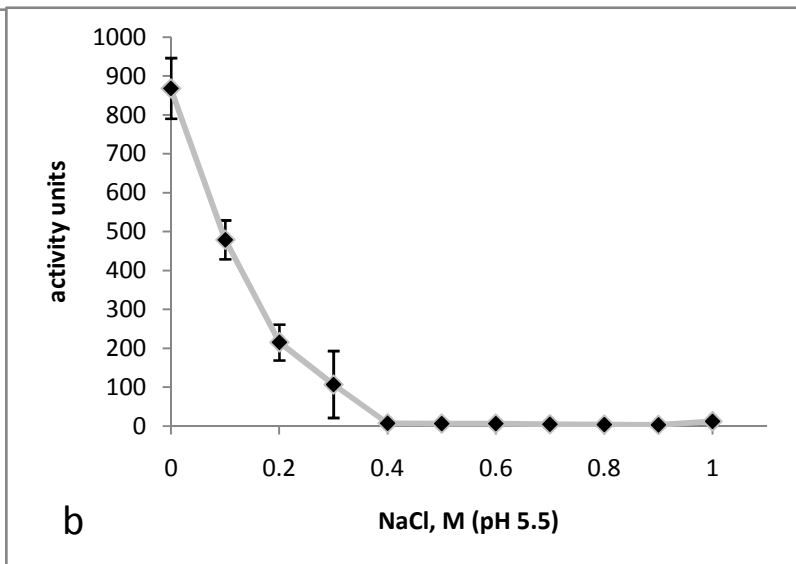

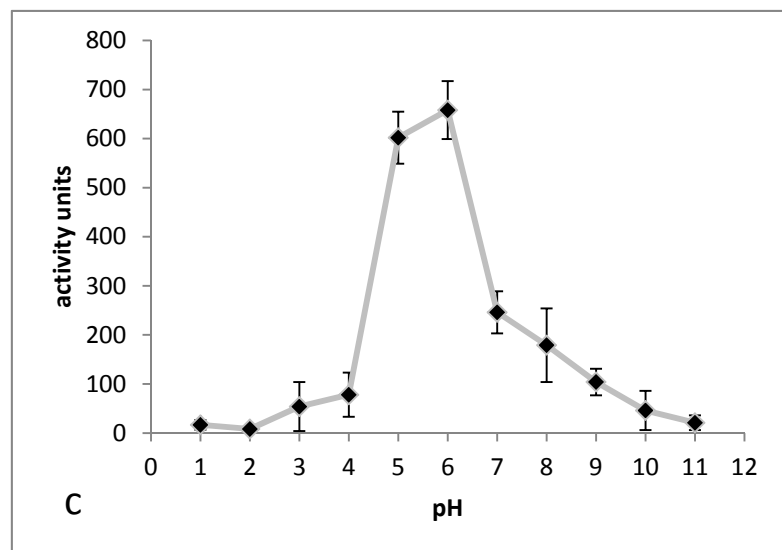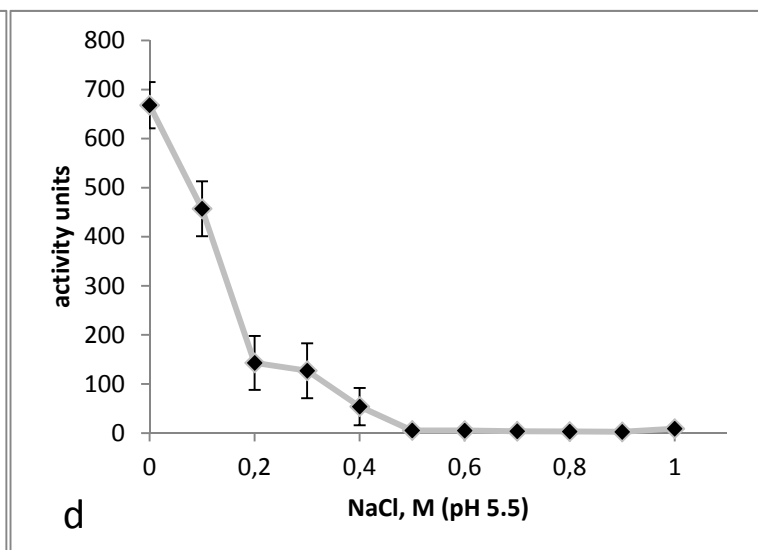

Supplement: Additional file 10: Figure S9. — Isopeptidase activity of mlDL isoforms. The effect of pH (a) and ionic strength (b) at pH 5.5 on the isopeptidase activity of mlDL-Ds1. The effect of pH (c) and ionic strength (d) at pH 5.5 on the isopeptidase activity of mlDL-Ds2. Isopeptidase activity was calculated according to formula (2). (n = 5). (PDF 100 kb) [file 12858_2015_56_MOESM10_ESM.pdf]

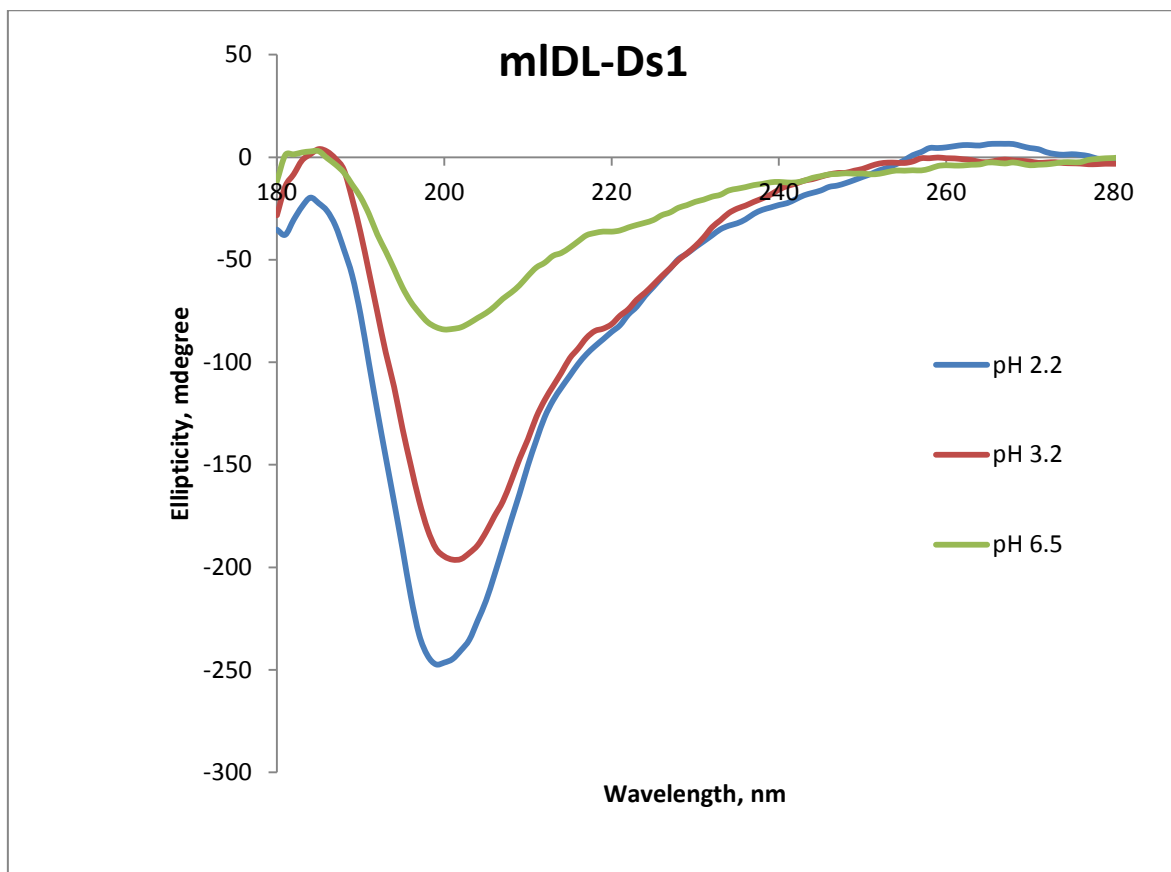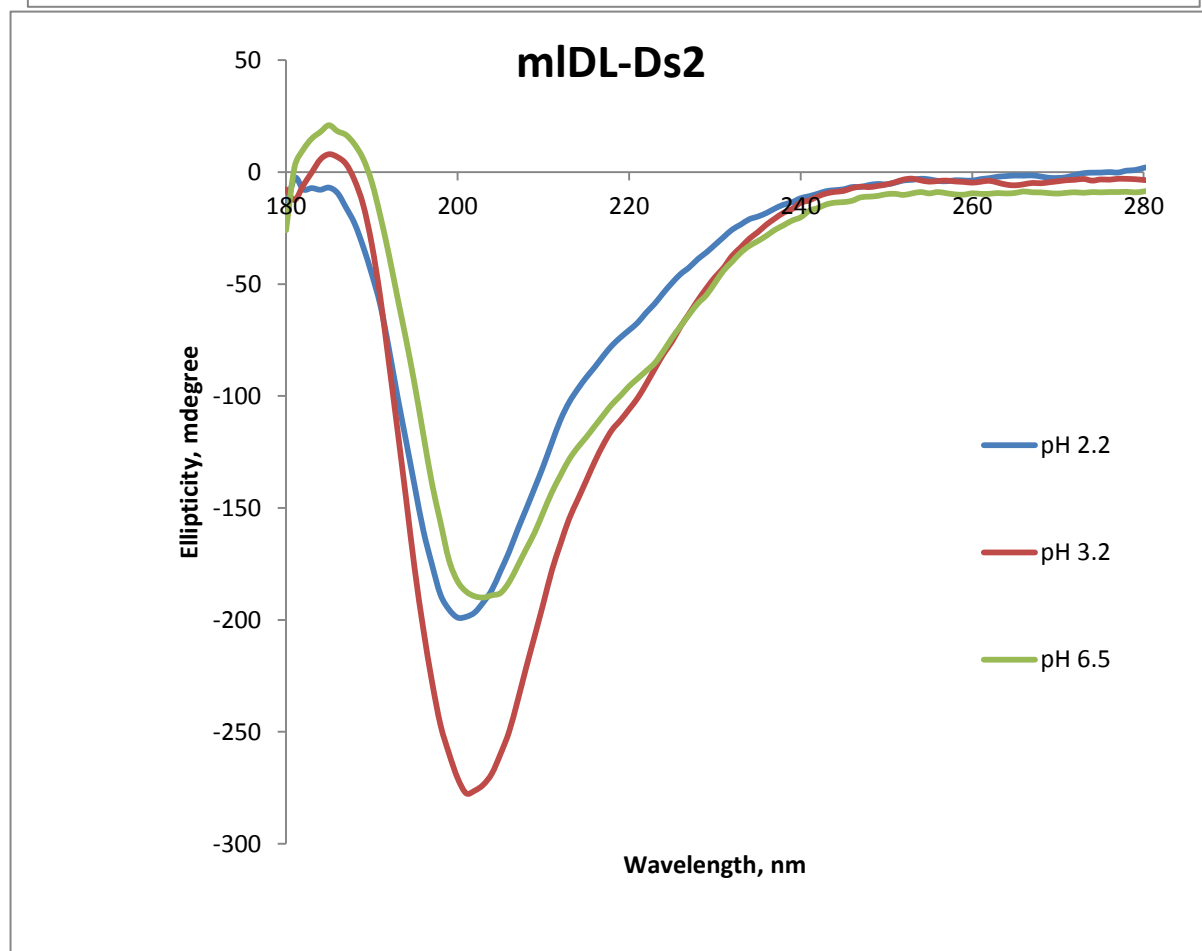

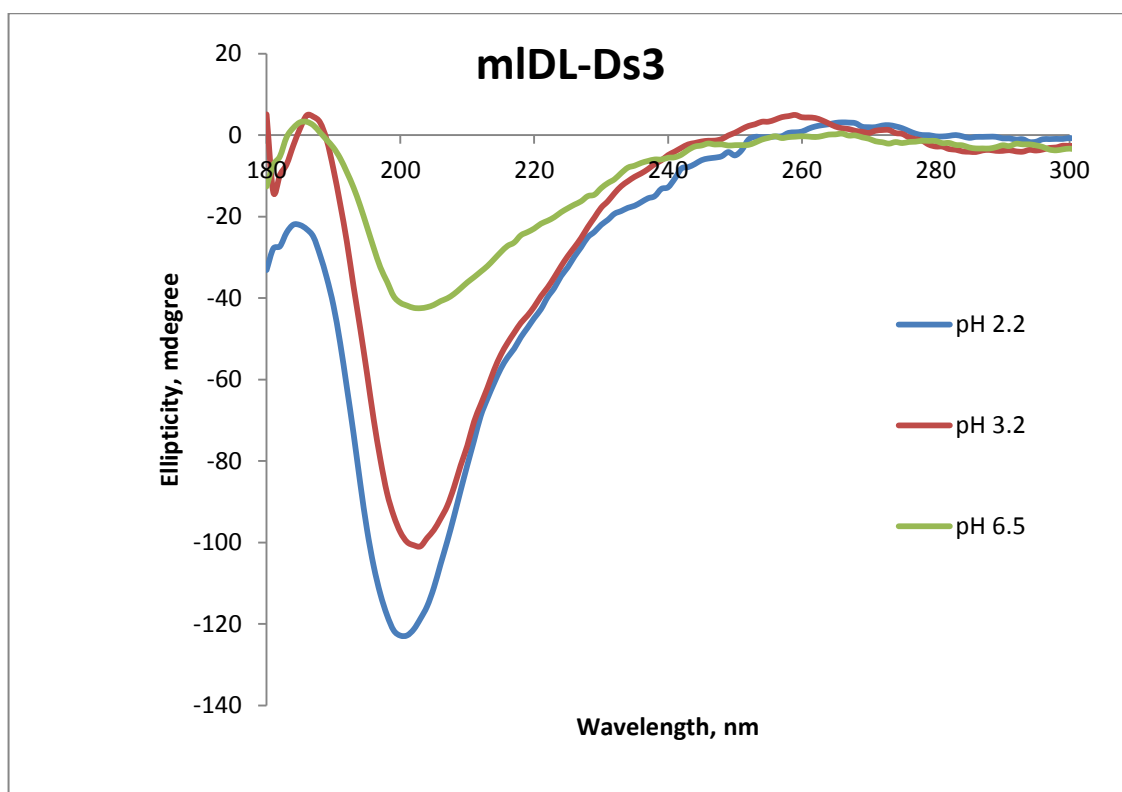

Supplement: Additional file 11: Figure S10. — CD spectra of mlDL isoforms at different pH conditions (2.2, 3.2, 6.5). (PDF 84 kb) [file 12858_2015_56_MOESM11_ESM.pdf]
