# Supplementary material for: A comparison of the enzymatic properties of three recombinant isoforms of thrombolytic and antibacterial protein—Destabilase-Lysozyme from medicinal leech
Source: BMC Biochem. 2015 Nov 21;16:27. doi: 10.1186/s12858-015-0056-3 (PMC4654880; doi:10.1186/s12858-015-0056-3)
Supplement: Additional file 3: — Scheme for de novo synthesising from oligonucleotides DNA fragments that encode the mlDL-Ds1, 2, 3 (optymized for E. coli ) and map of plasmid, encoding mlDL. Figure S2. Fragment of pET15MCS plasmid containing multicloning sites. PT7—promoter of late genes of bacteriophage T7; RBS—ribosome binding site; ATG—start codon; MCS—multiple cloning site; stop—stop codon; 6His—fragment encoding six histidines; tromb—fragment encoding the recognition site of thrombin. Figure S3. A map of the plasmid pET15/Dest. PT7—promoter of bacteriophage T7 late genes; dest—fragment encoding the mlDL isoforms; 6His—region encoding the hexa-histidine motive; stop—translation terminator; term—a transcription terminator; bla—beta-lactamase gene; pBR322ori—an origin of replication of plasmid pBR322; lacI—gene lacI of E. coli; tromb—sequence encoding the recognition site of thrombin. (PDF 119 kb) [file 12858_2015_56_MOESM3_ESM.pdf]

Scheme for de novo synthesising from oligonucleotides DNA fragments that encode the mlDL-Ds1, 2, 3 (optymized for E. coli).

The DNA fragment encoding mlDL-Ds1 designed for subsequent expression in *E. coli* was generated by enzyme-mediated extension of synthetic oligonucleotides. To this end, we prepared a mixture of the oligonucleotides Ds1-01 - Ds1-14 (Additional file 1: Table S1). The concentration of each oligonucleotide was 10 μM. One microlitre of the mixture was transferred into a tube filled with 20 μl of a solution containing 1× PCR-buffer, a mixture of dNTPs (the concentration of each dNTP was 0.2 mM) and 0.25 units of Taq-polymerase. The reaction mixture was incubated as follows: 95°C for 10 sec, 60°C for 10 sec, 72°C for 15 sec, for a total of 25 cycles. The obtained products of the reaction were used as matrices for the PCR with the Ds1-Sal and pDs1-Bam oligonucleotides.

For mlDL-Ds2 we prepared a mixture of the oligonucleotides DsEc-00 - DsEc-14. The obtained products of the reaction were used as matrices for the PCR with the DsEc-Sal and CrDes2 oligonucleotides.

For mlDL-Ds3 we prepared a mixture of the oligonucleotides dest3-1 - dest3-15. The obtained products of the reaction were used as matrices for the PCR with the dest3-sal and dest3-bam5 oligonucleotides.

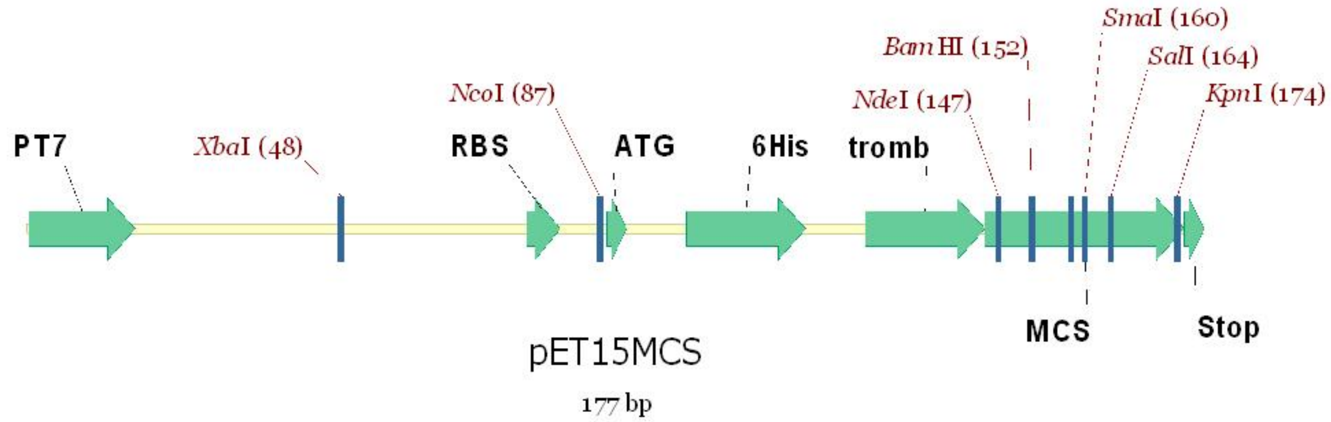

Figure S2. Fragment of pET15MCS plasmid containing multicloning sites. PT7 – promoter of late genes of bacteriophage T7; RBS - ribosome binding site; ATG - start codon; MCS – multiple cloning site; stop - stop codon; 6His - fragment encoding six histidines; tromb - fragment encoding the recognition site of thrombin.

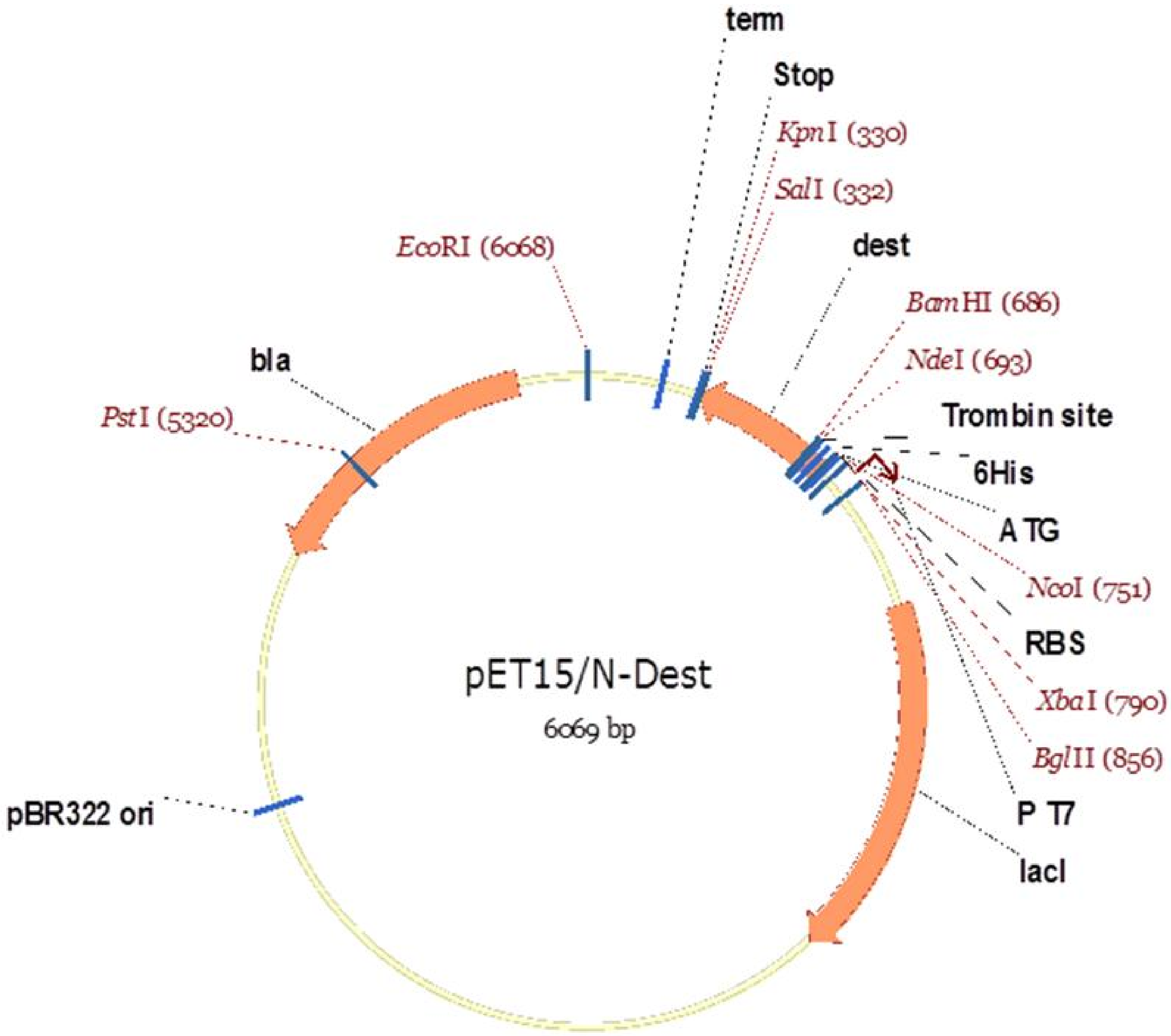

Figure S3. A map of the plasmid pET15/Dest. PT7 - promoter of bacteriophage T7 late genes; dest - fragment encoding the mlDL isoforms; 6His - region encoding the hexa-histidine motive; stop - translation terminator; term - a transcription terminator; bla - beta-lactamase gene; pBR322ori - an origin of replication of plasmid pBR322; lacI - gene lacI of E. coli; tromb - sequence encoding the recognition site of thrombin.
