# Supplementary material for: A comparison of the enzymatic properties of three recombinant isoforms of thrombolytic and antibacterial protein—Destabilase-Lysozyme from medicinal leech
Source: BMC Biochem. 2015 Nov 21;16:27. doi: 10.1186/s12858-015-0056-3 (PMC4654880; doi:10.1186/s12858-015-0056-3)
Supplement: Additional file 4: — Detailed scheme of isolating mlDL isoforms from inclusion bodies and their optimisation of renaturation by dialysis. mlDL-Ds1, 2, 3 were isolated using metal chelate affinity chromatography under denaturing conditions. Optimisation of mlDL isoforms accumulation in E. coli cells. We empirically selected the strains (8 strains), media (7 media) and cultivation schemes. Table S2. Accumulation levels of the recombinant mlDL isoforms in inclusion bodies from different E. coli strains. Accumulation levels are expressed in mg of protein per litre of culture (n = 3). Table S3. Accumulation levels of the mlDL in inclusion bodies from E. coli BL21(DE3)-gold in different culture media. Accumulation levels are expressed in mg of protein per litre of culture (i = 3). (PDF 64 kb) [file 12858_2015_56_MOESM4_ESM.pdf]

Detailed scheme of isolating mlDL isoforms. mlDL-Ds1, 2, 3 were isolated using metal chelate affinity chromatography under denaturing conditions.
The transformed *E. coli* cells were pelleted by centrifugation at 5000×g for 15 min. Next, the pellets were resuspended in lysis buffer (20 mM Tris-Cl, 100 mM NaCl, 0.1% Triton X-100) and disrupted using a sonicator (Branson 250 Sonifier; Branson, USA) at 22 kHz for 10 min. The solutions were then centrifuged at 20000×g for 15 min. The resulting pellets were resuspended in a solution of 1% Triton X-100 and precipitated by centrifugation at 20000×g for 20 min. The pellets were then washed with detergent 3 times followed by a single washing with PBS. The washed inclusion bodies were solubilised in at least 10 ml of 20 mM Na-phosphate buffer (pH 7.5) containing 8 M urea, 10 mM imidazole, 0.5 M NaCl and 0.1% β-mercaptoethanol per 1 litre of culture. The solutions were incubated for 1 hour at 50°C. The precipitates were separated by centrifugation at 15000×g for 15 min. The supernatants were collected and filtered through the filters with a pore diameter of 0.45 μm (Sartorius).
The resulting solutions were placed in a chromatographic column (XK-16/20; GE Healthcare, USA) filled with 10 ml of sorbent Ni Sepharose High Performance (GE Healthcare, USA) and equilibrated with the same buffer. After application, the columns were washed with at least 50 ml of starting buffer and then washed again with 20 mM Na-phosphate buffer (pH 7.5) containing 8 M urea, 40 mM imidazole, and 0.5 M NaCl. Subsequently, the protein was eluted with 20 mM Na-phosphate buffer (pH 7.4) containing 8 M urea, 0.5 M NaCl, and 500 mM imidazole. The flow rate of the buffer was 1 ml/min. The process and collection of the fractions was monitored by measuring the eluate absorbency at 280 nm. The target proteins were purified using a chromatograph AKTA FPLS (GE Healthcare, USA). mlDL isoforms were determined in the fractions by Laemmli SDS-PAGE.

Optimisation of mlDL isoforms accumulation in E. coli cells. We empirically selected the strains (8 strains), media (7 media) and cultivation schemes.
To increase the accumulation levels of the mlDL isoforms, we tested several culture media (LB, LB2x, SOB, M9+Glc, 2YT, TB, SB) and strains of *E. coli* (origami (DE3), origami (DE3) pLysS, BLR, BLR pLysS, BL21 (DE3), BL21 (DE3) pLysS, BL21 (DE3) gold, B834). We also tested two transcriptional inducers (lactose and IPTG). We tested several cultivation schemes by varying the incubation temperature and optical density of the culture immediately prior to induction.
E. coli cells were cultivated in a shaker-incubator at 180 rev/min. Fractions of insoluble proteins were isolated from the resulting bacterial cultures and analysed by Laemmli SDS-PAGE.
1. Selection of the producer strain. The strains were transformed with the plasmids carrying genes encoding mlDL isoforms. The cells were grown in LB medium containing 10 mM lactose for 18 hours. Cultivation was performed at 25°C and 37°C for each strain. The levels of accumulation of the recombinant mlDL isoforms are shown in Additional file 7: Table S2. For further work, we selected *E. coli* BL21 (DE3) gold.
2. Selection of culture medium. The transformed *E. coli* strain BL21 (DE3) was grown in several media types containing 10 mM lactose at 37°C for 18 h. The levels of

accumulation of the recombinant destabilase isoforms are shown in Additional file 8: Table S3. For further work, we selected the SB medium.
3. Selection of the cultivation scheme. We tested four cultivation schemes:
1) Individual colonies were inoculated into the medium containing the transcriptional inducer and incubated for 18 h and 24 h.
2) Individual colonies were inoculated into the medium without the transcriptional inducer and cultured at 37°C overnight. The overnight culture was added to the fresh medium at a dilution of 1:20 and grown until OD_{600} = 0.8. Next, the transcriptional inducer was added, and the cells were grown for an additional 4 h, 16 h and 24 h.
3) Individual colonies were inoculated into the medium without the transcriptional inducer and cultured at 37°C overnight. The overnight culture was added to the fresh medium at a dilution of 1:20 and grown until OD_{600} = 2.0. Next, the transcriptional inducer was added, and the cells were grown for an additional 4 h, 16 h and 24 h.
4) Individual colonies were inoculated into the medium without the transcriptional inducer and cultured at 37°C overnight. Next, the cells were pelleted by centrifugation and resuspended in the same volume of fresh medium. After incubation for 1 h, the transcriptional inducer was added, and the cells were grown for an additional 4 h, 16 h and 24 h.
Each cultivation scheme was tested by varying the concentration of transcriptional inducers (10 mM and 1 mM in the case of lactose and 1 mM and 0.1 mM in the case of IPTG). In all cases, the incubations were performed at 25°C and 37°C.
We selected the following cultivation scheme: single colonies of *E. coli* were inoculated into the SB medium without the transcriptional inducer and cultured at 37°C overnight. The overnight culture was added to the fresh medium at a dilution of 1:20 and grown until OD_{600} = 0.8. Next, lactose was added at a concentration of 10 mM, and the cells were grown at 37°C for an additional 6-7 h.

Table S2. Accumulation levels of the recombinant mlDL isoforms in inclusion bodies from different E. coli strains. Accumulation levels are expressed in mg of protein per litre of culture. (n=3)

	origami	OrigamipLysS	BLR	BLR pLysS	BL21-gold	BL21	BL21 pLysS	B834
37°C	0	0	0	0	13	3	6	9
25°C	27	0	0	0	23	20	23	23

Table S3. Accumulation levels of the mlDL in inclusion bodies from E. coli BL21(DE3)-gold in different culture media. Accumulation levels are expressed in mg of protein per litre of culture. (n=3)

LB	LB2x	SOB	M9	2YT	TB	SB
30	40	0	0	27	40	37

Optimisation of renaturation by dialysis of the mlDL isoforms isolated from inclusion bodies.

The dialysis of purified mlDL was carried out for 18 h. Next, the supernatant was separated using centrifugation. The precipitate was dissolved in the same volume of 1% SDS. The samples were analysed using denaturing PAGE electrophoresis. We used the following buffers: 20 mM sodium phosphate buffer at the pH values 5.0, 6.0, 7.0, 8.0 and 9.0, and 20 mM Tris-HCl at the same pH values. For further investigation, 20 mM sodium phosphate buffer at pH 5.0 was used to obtain the highest yield of soluble protein. Traditionally used additives, such as glutathione, 2-mercaptoethanol, DTT and PEG, were found to reduce the yield of soluble protein.

Furthermore, we analysed the effect of both temperature and protein concentration on the refolding efficiency. We found that a low initial protein concentration resulted in a decrease in the amount of the precipitated protein. It has also been found that refolding occurs better at 4°C than at higher temperatures.
