# Supplementary material for: A comparison of the enzymatic properties of three recombinant isoforms of thrombolytic and antibacterial protein—Destabilase-Lysozyme from medicinal leech
Source: BMC Biochem. 2015 Nov 21;16:27. doi: 10.1186/s12858-015-0056-3 (PMC4654880; doi:10.1186/s12858-015-0056-3)
Supplement: Additional file 5: Figure S4. — MALDI-TOF analysis of mlDL isoforms. (PDF 388 kb) [file 12858_2015_56_MOESM5_ESM.pdf]

Analysis of the protein tryptic charts of additional bands in mlDL sample by MALDI-TOF mass-spectrometry.

mlDL-Ds1 main band with MW near 16 kDa.

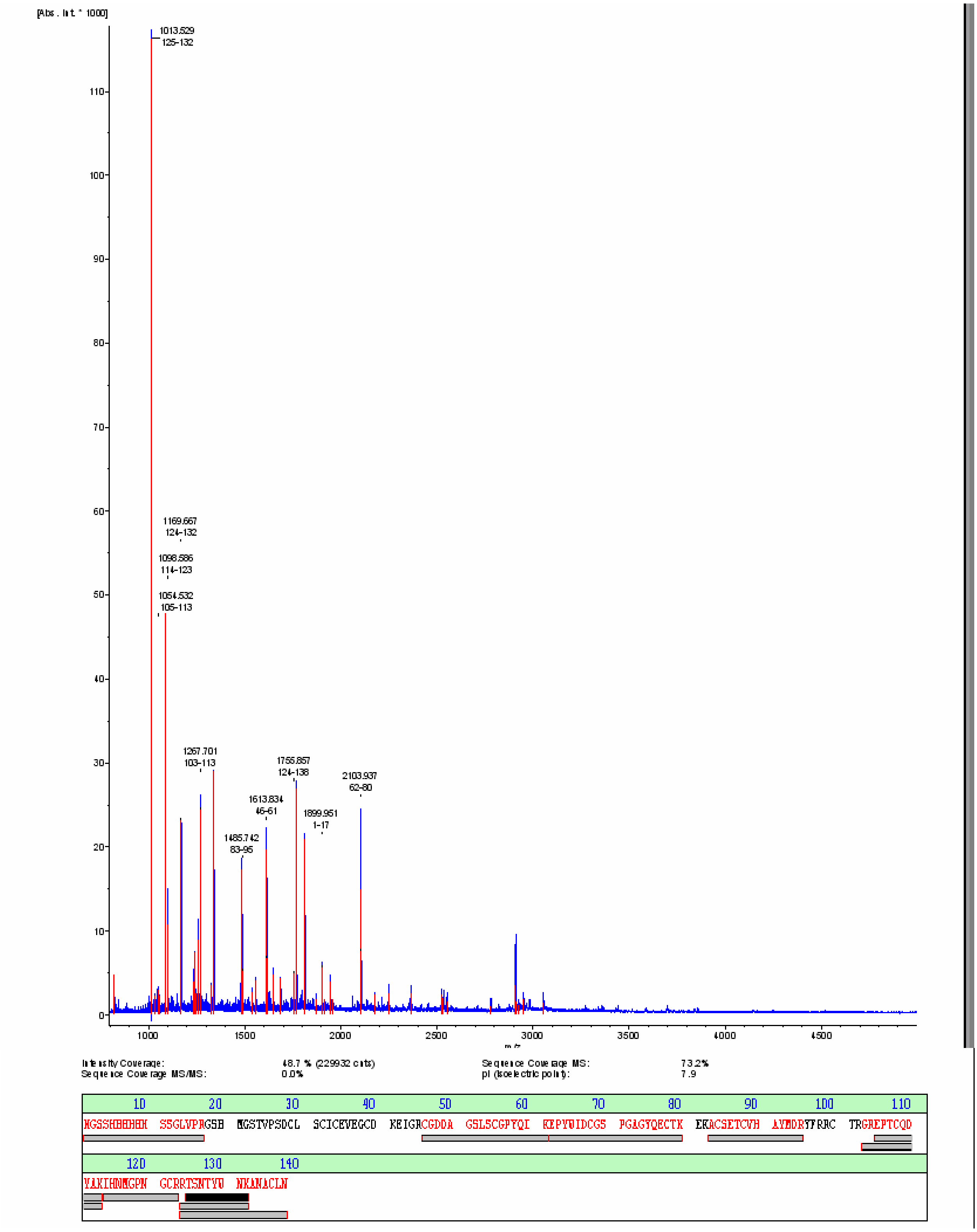

mlDL-Ds1 additional band with MW near 28 kDa.

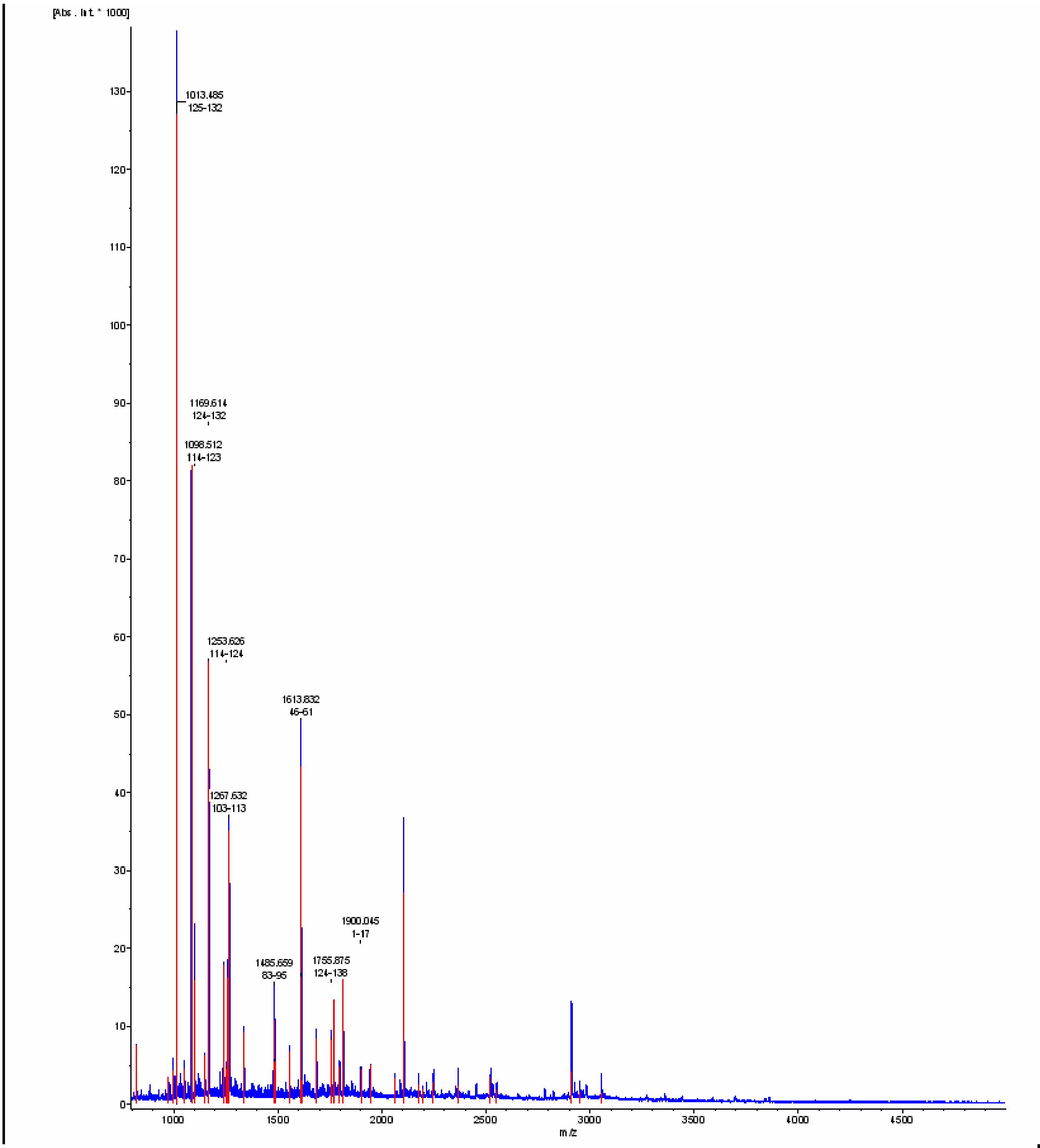

Intensity Coverage:	44.7 % (294611 cnts)	Sequence Coverage MS:	59.4%
Sequence Coverage MS/MS:	0.0%	pI (isoelectric point):	7.9

10	20	30	40	50	60	70	80	90	100	110
MGSSHHHHHH	SSGLVPRGSH	MGSTVPSDCL	SCICEVEGCD	KEIGRCGDDA	GSLSCGPYQI	KEPYVIDCGS	PGAGYQECTK	EKACSETCVH	AYMDRYFRAC	TRGREPTCQD
**120**	**130**	**140**								
YAKIHNMGPN	GCRRTSNTYV	NKANACLN								

mlDL-Ds1 additional band with MW near 44 kDa.

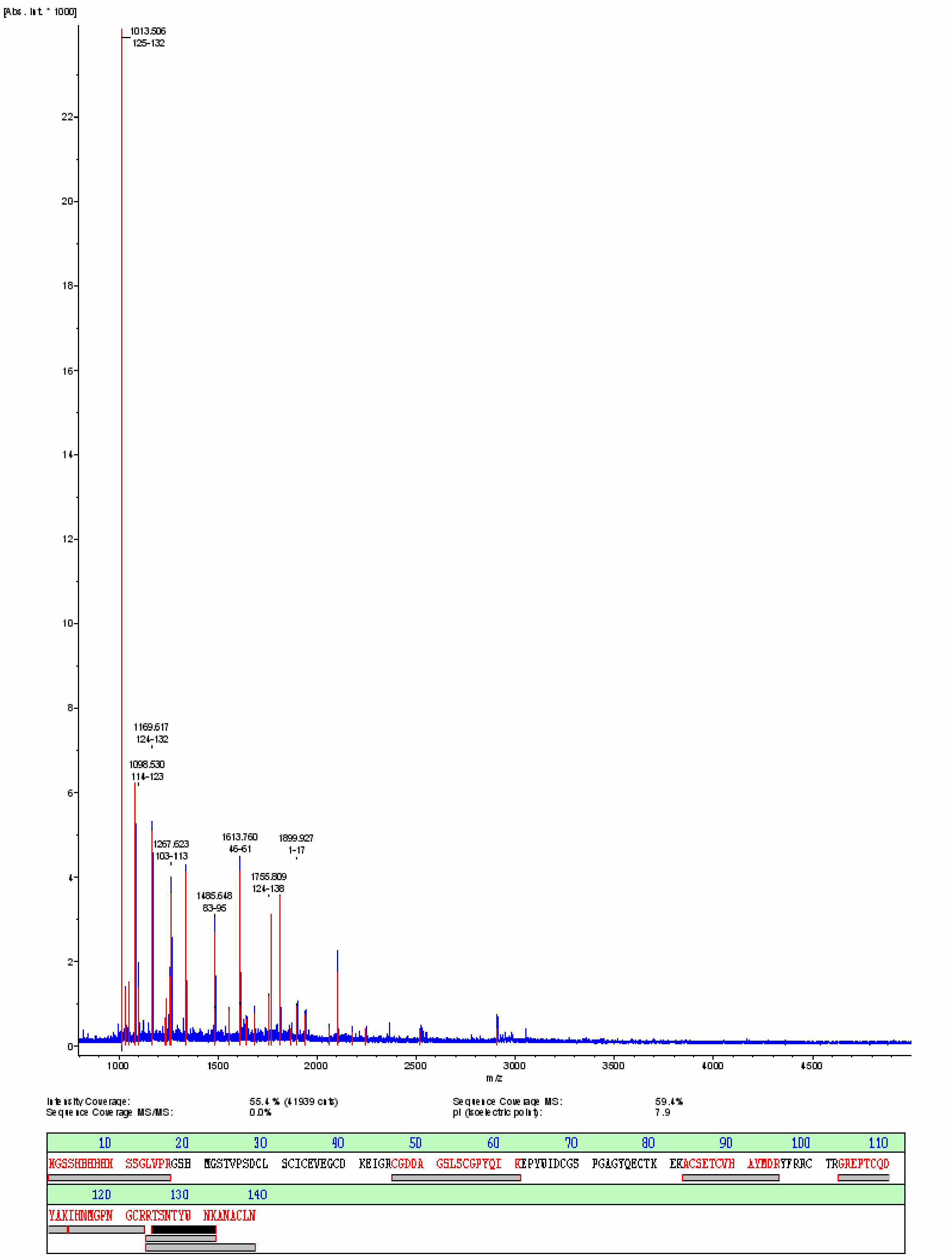

mIDL-Ds2 main band with MW near 16 kDa.

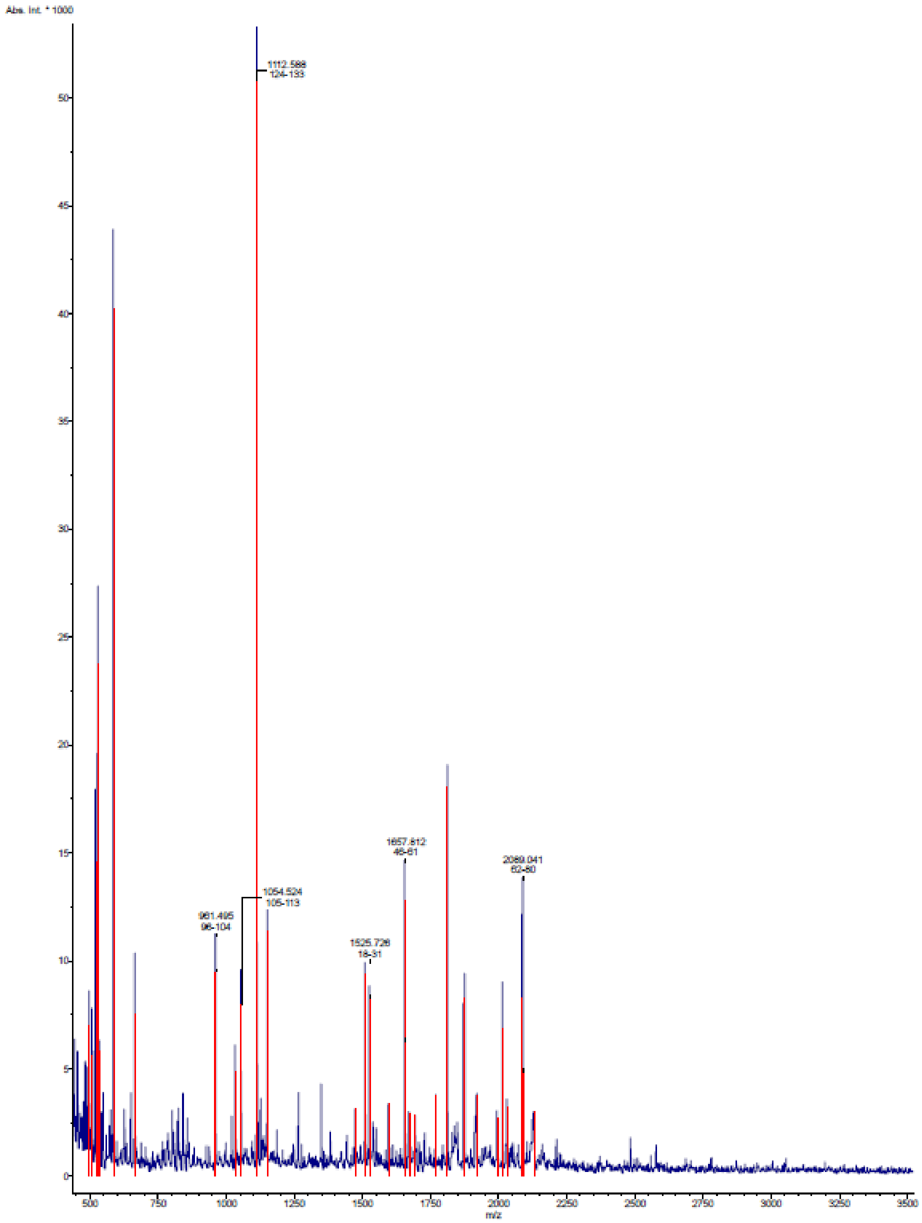

Intensity Coverage:	31.6 % (95419 cnts)	Sequence Coverage MS:	55.4%
Sequence Coverage MS/MS:	0.0%	pI (isoelectric point):	10.4

10 20 30 40 50 60 70 80 90 100 110 120 130 140

MGSSHHHHHH SSGLVPRGSH MGSQFTDSCL RCICKVEGCD SQIGKCGMDV GSLSCGPYQI KKPYWIDCGK PGGGYESCTK NKACSETCVR AYMKRYGTFC TGGRTPTCQD YARIHNGGPR GCKSSATVGY WNKVQKCLR

mlDL-Ds2 additional band with MW near 28 kDa.

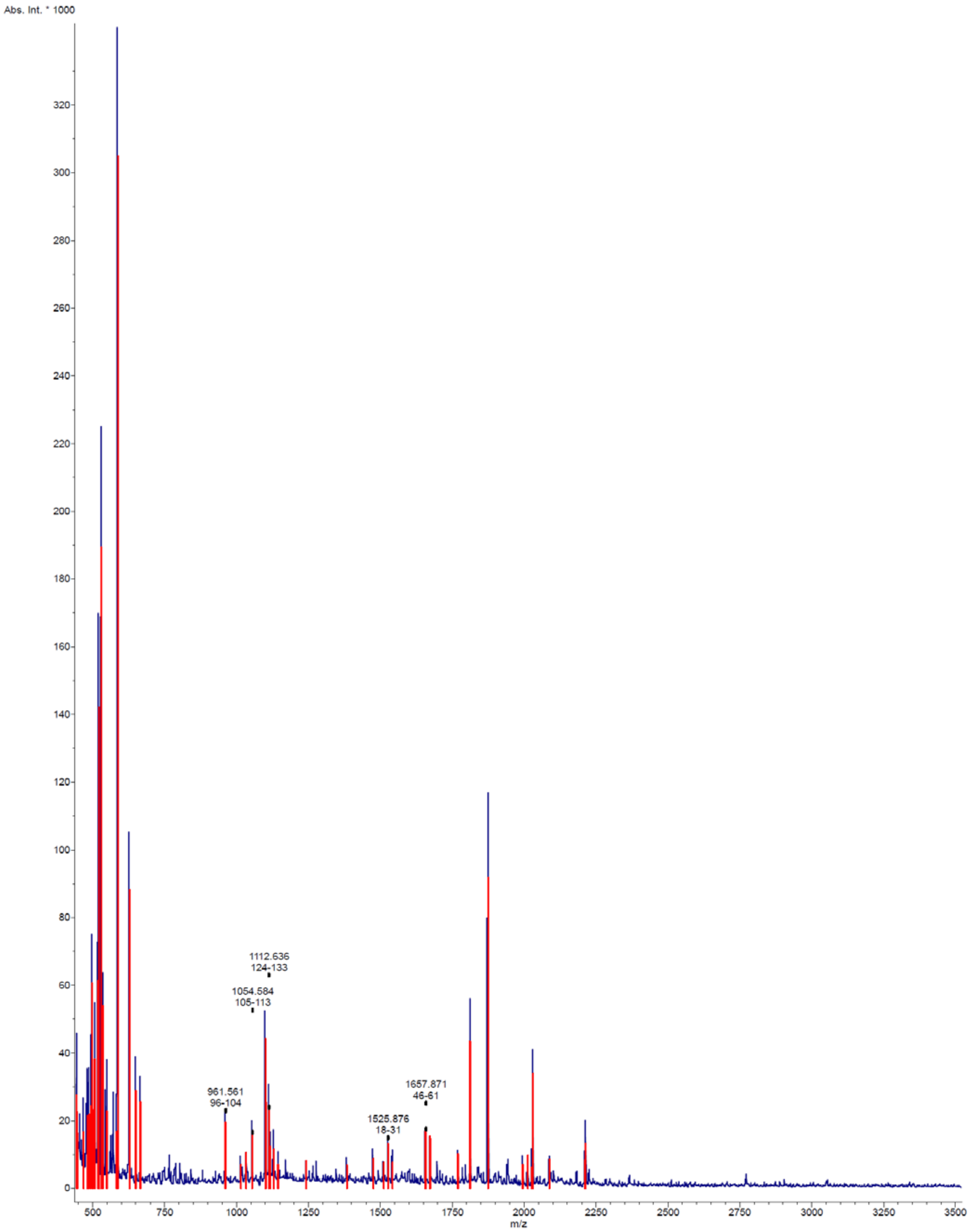

Intensity Coverage:	5.0 % (87055 cnts)	Sequence Coverage MS:	41.7%
Sequence Coverage MS/MS:	0.0%	pI (isoelectric point):	10.4

10 20 30 40 50 60 70 80 90 100 110 120 130 140

MGSSHHHHHH SSGLVPRGSH MGSQFTDSCL RCICKVEGCD SQIGKCGMDV GSLSCGPYQI KKPYWIDCGK PGGGYESCTK NKACSETCVR AYMKRYGTFC TGGRIPTCQD YARIHNGGPR GCKSSATVGY WNKVQKCLR

mIDL-Ds2 additional band with MW near 44 kDa.

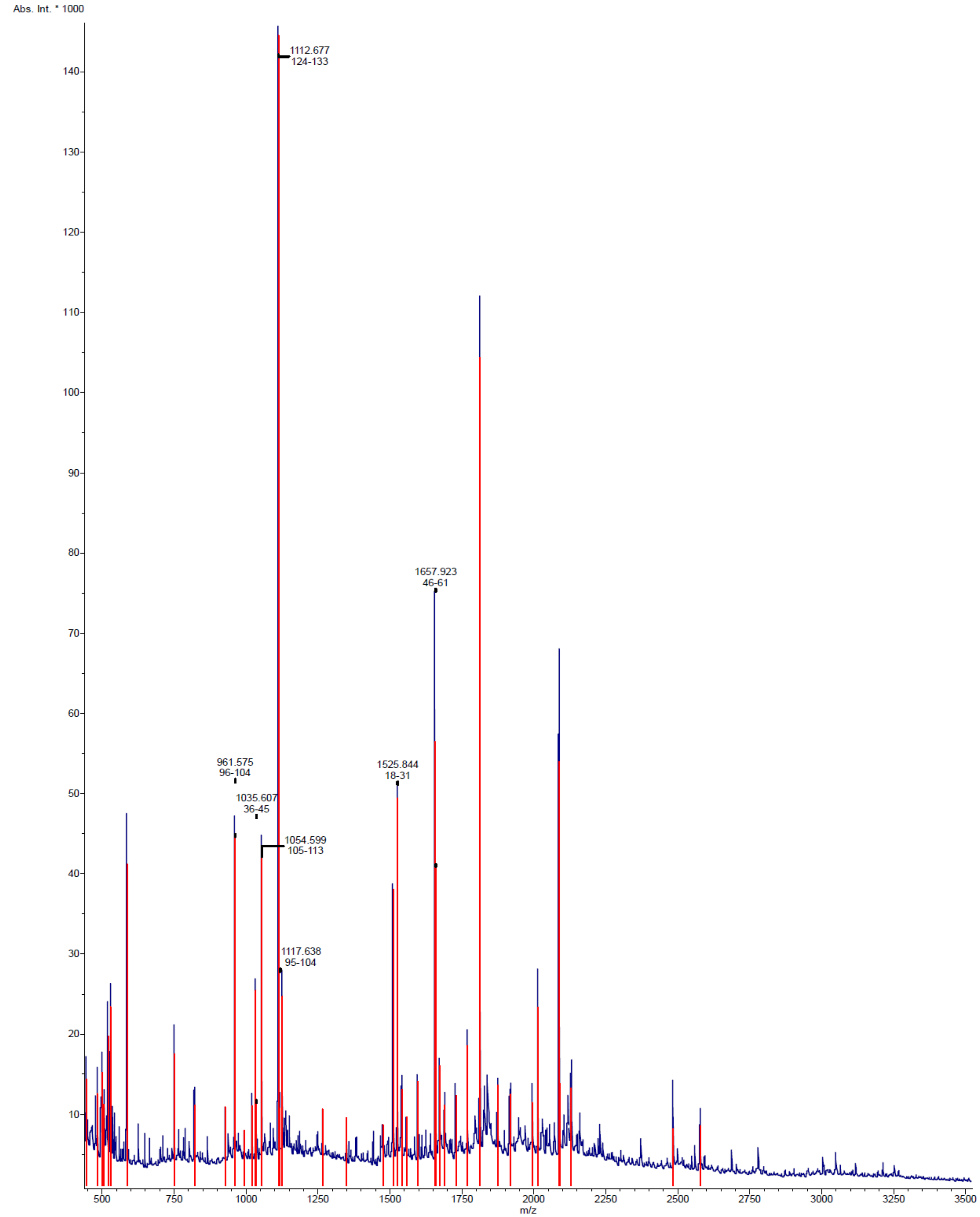

Intensity Coverage:	33.5 % (351753 cnts)	Sequence Coverage MS:	49.6%
Sequence Coverage MS/MS:	0.0%	pI (isoelectric point):	10.4

10 20 30 40 50 60 70 80 90 100 110 120 130 140

MGSSHHHHHH SSGLVPRGSH MGSQFTDSCL RCICKVEGCD SQIGKCGMDV GSLSCGPYQI KKPYWIDCGK PGGGYESCTK NKACSETCVR AYMKRYGTFC TGGRTPTCQD YARIHNGGPR GCKSSATVGY WNKVQKCLR

mlDL-Ds3 main band with MW near 16 kDa.

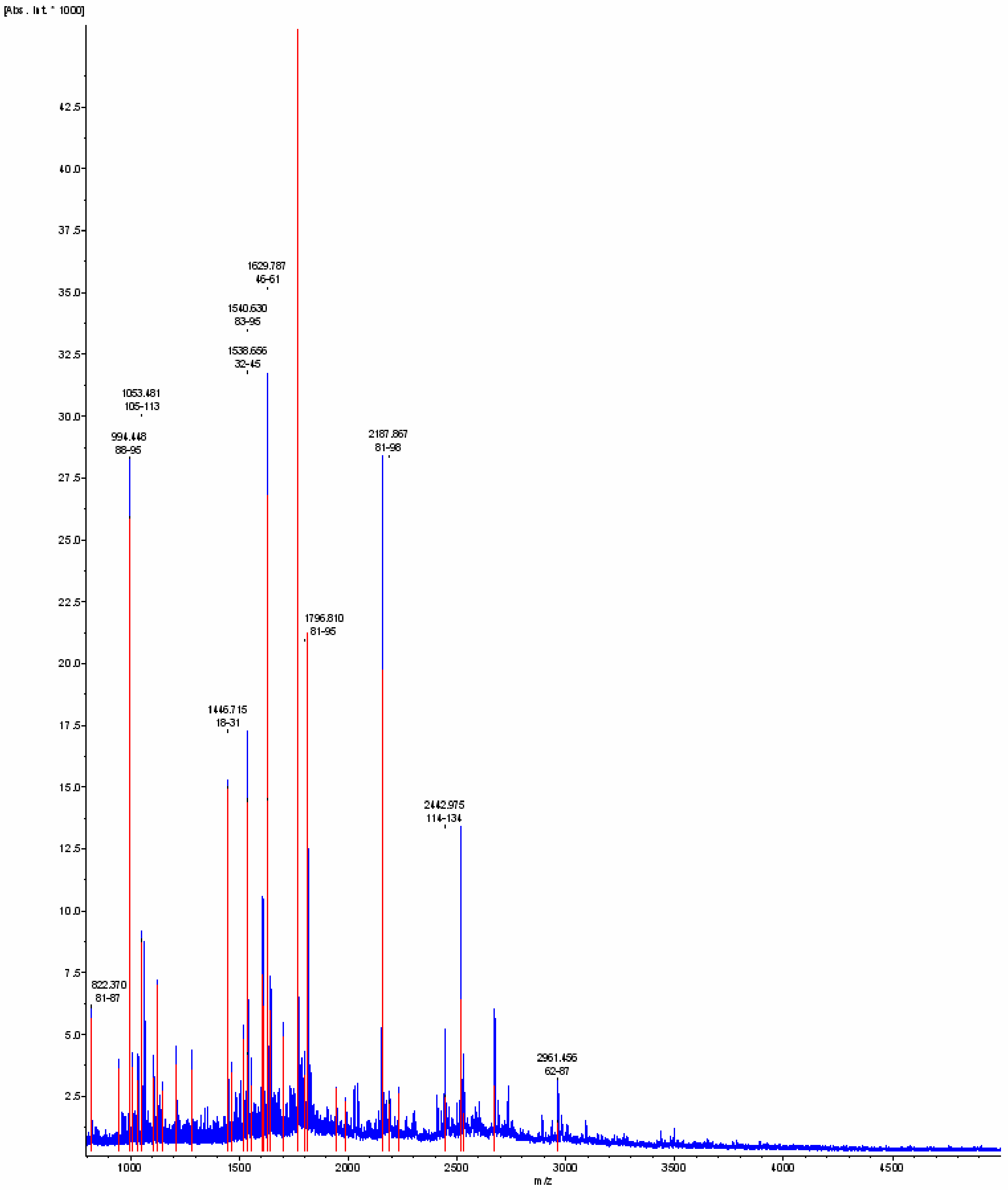

Intensity Coverage:	37.6 % (115376 cnts)	Sequence Coverage MS:	81.2%
Sequence Coverage MS/MS:	0.0%	pI (isoelectric point):	9.8

10	20	30	40	50	60	70	80	90	100	110
MGSSHHHHHH	SSGLVPRGSH	MGSTVPSDCL	RCICQVEGCN	NEIGRCGMDA	GSLSCGPYQI	KEPYWIDCGR	PGGGYQQCTK	EKACSERCVH	AYMDRYARRC	TGGRQPTCQD

120	130	140
YAKIHNGGPN	GCQSSNNHYW	DNVRRCLG

mlDL-Ds3 additional band with MW near 28 kDa.

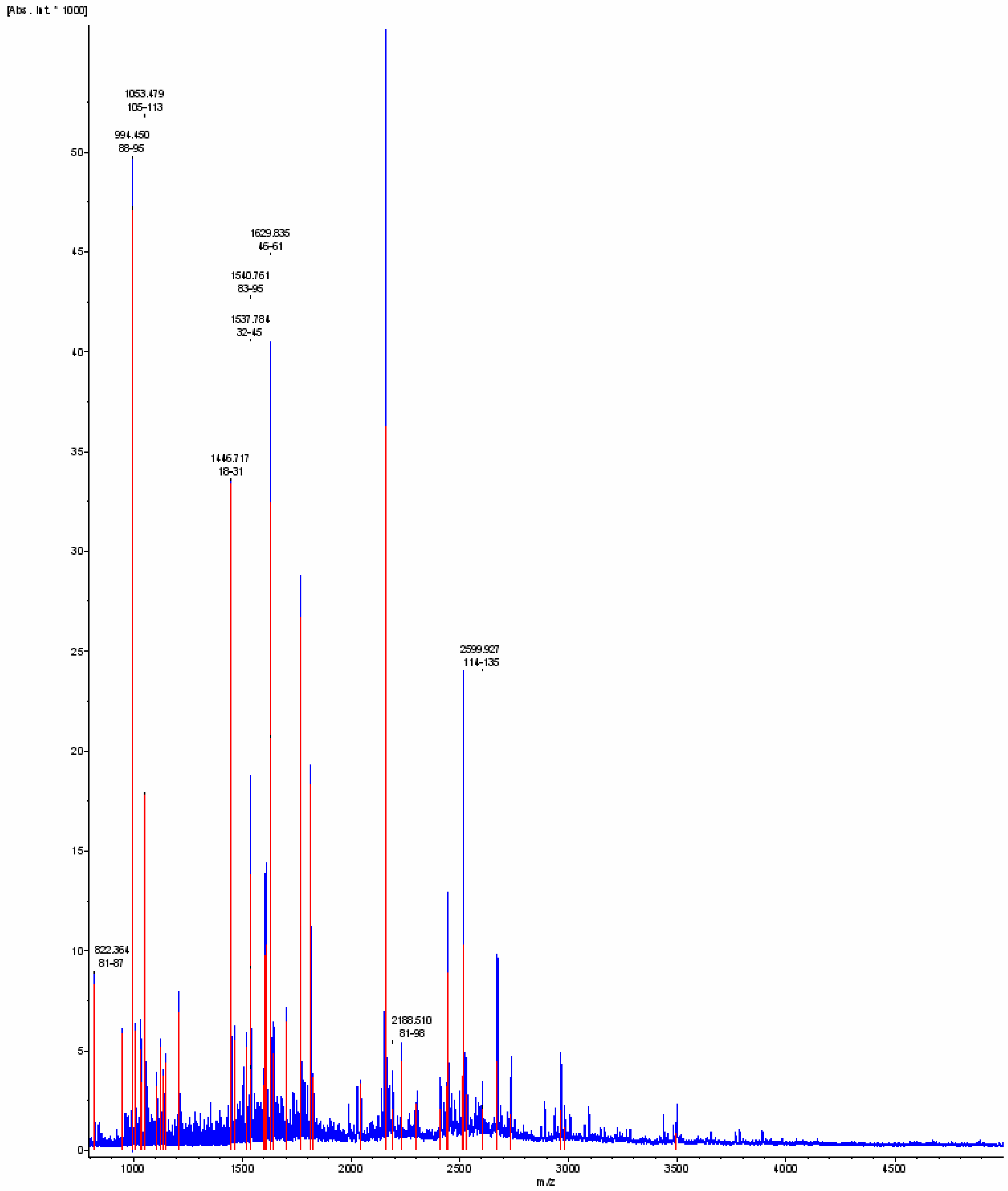

Intensity Coverage: 37.5 % (164463 cnts)
Sequence Coverage MS/MS: 0.0%
Sequence Coverage MS: 68.1%
pI (isoelectric point): 9.8

10	20	30	40	50	60	70	80	90	100	110
MGSSHHHHHH	SSGLVPRGSH	MGSTVPSDCL	RCICQVEGCN	NEIGRCGEDA	GSLSCGPYQI	KEPYVIDCGR	PGGGYQQCTK	EKACSERCVH	AYMDRYARRC	TGRQPTCQD

120	130	140
YAKIHNMGPN	GCQSSNNHYV	DNVARCLG

mlDL-Ds3 additional band with MW near 44 kDa.

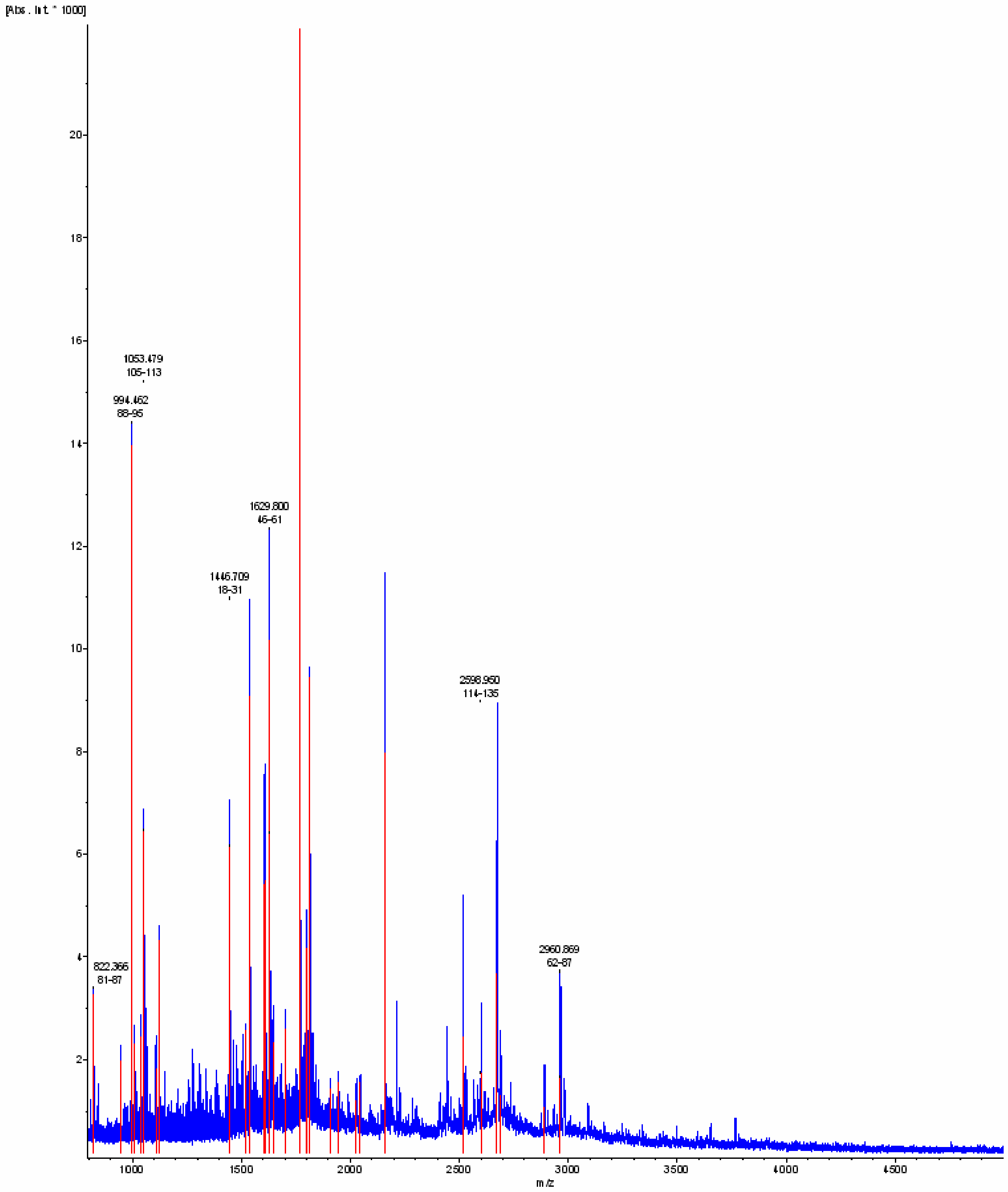

Intensity Coverage: 27.4 % (42268 cnts)
Sequence Coverage MS/MS: 0.0%
Sequence Coverage MS: 68.8%
pI (isoelectric point): 9.8

```
        10         20         30         40         50         60         70         80         90        100        110
MGSSHHHHHH SSGLVPRGSH MGSTVPSDCL RCICQVEGCN NEIGRCGEDA GSLSCGPYQI KEPYVIDCGR PGGGYQQCTK EKACSERCVH AYMDRYARRC TGGRQPTCQD
       120        130        140
YAKIHNMGPN GCQSSNNEYV DNVRRCLG
```
